# Cannabinoids as Antibacterial Agents: A Systematic and Critical Review of In Vitro Efficacy Against *Streptococcus* and *Staphylococcus*

**DOI:** 10.3390/antibiotics13111023

**Published:** 2024-10-30

**Authors:** Dhakshila Niyangoda, Myat Lin Aung, Mallique Qader, Wubshet Tesfaye, Mary Bushell, Fabian Chiong, Danny Tsai, Danish Ahmad, Indira Samarawickrema, Mahipal Sinnollareddy, Jackson Thomas

**Affiliations:** 1 Faculty of Health, University of Canberra, Canberra, ACT 2617, Australia; dhakshila.niyangoda@canberra.edu.au (D.N.); mary.bushell@canberra.edu.au (M.B.); 2 Department of Pharmacy, Faculty of Allied Health Sciences, University of Peradeniya, Peradeniya 20400, Sri Lanka; 3 Institute for Tuberculosis Research, Department of Pharmaceutical Sciences, University of Illinois Chicago, Chicago, IL 60612, USA; mallique@uic.edu; 4 School of Pharmacy, Faculty of Health and Behavioural Sciences, The University of Queensland, St Lucia, QLD 4072, Australia; 5 Department of Infectious Diseases, The Canberra Hospital, Garran, ACT 2605, Australia; fabian.chiong@act.gov.au; 6 School of Medicine and Psychology, Australian National University, Canberra, ACT 2601, Australia; danish.ahmad@anu.edu.au; 7 Flinders Health and Medical Research Institute, College of Medicine and Public Health, Flinders University, Alice Springs, NT 0870, Australia; d.tsai@uq.edu.au; 8 University of Queensland Centre for Clinical Research, The University of Queensland, Herston, QLD 4029, Australia; 9 Pharmacy Department, Alice Springs Hospital, Central Australian Region Health Service, Alice Springs, NT 0870, Australia; 10 Strategy Coaching and Research Consulting Pty Ltd., O’Malley, ACT 2606, Australia; info@indirasam.com; 11 Clinical Pharmacology and Pharmacometrics, AbbVie Inc., 1 North Waukegan Road, North Chicago, IL 60064, USA; mahipal.sinnollareddy@abbvie.com

**Keywords:** antibacterial, medicinal cannabis, cannabinoids, *Streptococcus*, *Staphylococcus*, infection, antimicrobial resistance, skin infections, dermatological conditions, infectious skin diseases

## Abstract

Background: Two major bacterial pathogens, *Staphylococcus aureus* and *Streptococcus pyogenes*, are becoming increasingly antibiotic-resistant. Despite the urgency, only a few new antibiotics have been approved to address these infections. Although cannabinoids have been noted for their antibacterial properties, a comprehensive review of their effects on these bacteria has been lacking. Objective: This systematic review examines the antibacterial activity of cannabinoids against *S. aureus*, including methicillin-resistant *S. aureus* (MRSA) and vancomycin-resistant *S. aureus* (VRSA) strains, and *S. pyogenes*. Methods: Databases, including CINAHL, Cochrane, Medline, Scopus, Web of Science, and LILACS, were searched. Of 3510 records, 24 studies met the inclusion criteria, reporting on the minimum inhibitory concentration (MIC) and minimum bactericidal concentration of cannabinoids. Results: Cannabidiol (CBD) emerged as the most effective cannabinoid, with MICs ranging from 0.65 to 32 mg/L against *S. aureus*, 0.5 to 4 mg/L for MRSA, and 1 to 2 mg/L for VRSA. Other cannabinoids, such as cannabichromene, cannabigerol (CBG), and delta-9-tetrahydrocannabinol (Δ9-THC), also exhibited significant antistaphylococcal activity. CBD, CBG, and Δ9-THC also showed efficacy against *S. pyogenes*, with MICs between 0.6 and 50 mg/L. Synergistic effects were observed when CBD and essential oils from *Cannabis sativa* when combined with other antibacterial agents. Conclusion: Cannabinoids’ antibacterial potency is closely linked to their structure–activity relationships, with features like the monoterpene region, aromatic alkyl side chain, and aromatic carboxylic groups enhancing efficacy, particularly in CBD and its cyclic forms. These results highlight the potential of cannabinoids in developing therapies for resistant strains, though further research is needed to confirm their clinical effectiveness.

## 1. Introduction

*Staphylococcus aureus* and *Streptococcus pyogenes* (Group A *Streptococcus*, GAS) are two of the most dangerous and lethal Gram-positive pathogens globally, responsible for a wide range of infections [1,2,3,4,5,6,7]. In 2019, *S. aureus* was linked to approximately 1.1 million deaths, while GAS caused 0.2 million [2]. While these bacteria have distinct pathogenic characteristics, they are frequently discussed together due to their significant roles as the primary causes of bacterial skin and soft tissue infections (SSTIs), where they often co-infect [8,9].

The management of SSTIs is becoming increasingly complicated by antimicrobial resistance (AMR), which poses a significant challenge for both *S. aureus* and *S. pyogenes* [10,11,12,13,14,15,16]. *S. aureus* is increasingly developing resistance to multiple frontline antibiotics, including amoxicillin–clavulanate, cephalexin, cephalosporins, clindamycin, dicloxacillin, flucloxacillin, and penicillin, which are commonly recommended for the treatment of SSTIs [10,17,18,19,20,21,22]. In 2019, antibiotic-resistant *S. aureus* was responsible for an estimated 178,000 deaths globally, with methicillin-resistant *S. aureus* (MRSA) accounting for approximately 100,000 of these fatalities [21]. Alarmingly, MRSA is progressively acquiring multi-drug resistance, including resistance to several commonly used anti-MRSA antibiotics, such as vancomycin, daptomycin, ceftaroline, and linezolid [23,24,25,26,27,28]. Similarly, GAS has exhibited rising resistance to a range of antibiotics, including penicillin (0–4.2%), clindamycin (0.8–19%), macrolides (0.5–95%), trimethoprim–sulfamethoxazole (1.4–83%), and tetracyclines (14–61%), over the past decade [11,12,19,29,30,31,32]. In 2019, drug-resistant GAS was attributed with 3630 deaths worldwide [21]. Although GAS is generally susceptible to beta-lactam antibiotics, alternative treatments are needed for penicillin-allergic patients and for when resistance is reported [19,29,30].

Given the rise in AMR, co-infection of SSTIs by *S. aureus* and GAS, and the limited efficacy of conventional treatments, there is an urgent and critical need to explore alternative therapeutic options that can target both *S. aureus* and *S. pyogenes* effectively [33]. Recent reviews have highlighted the potential of cannabinoids, particularly cannabidiol (CBD), as promising alternatives due to their antibacterial properties [34,35,36]. Pre-clinical studies prove the antibacterial efficacy of cannabinoids, especially against *S. aureus* [37,38,39,40]. In addition to their antibacterial properties, cannabinoids possess therapeutic attributes such as anti-pruritic, anti-inflammatory, analgesic, and wound-healing effects, which enhance their potential in treating SSTIs [41,42,43]. Notably, a phase II clinical trial of CBD as a topical agent for treating *S. aureus* nasal colonization has been completed, further supporting its significance [44]. A recent systematic review of preclinical and clinical studies further underscored the wound-healing and antimicrobial benefits of cannabinoids in SSTIs, highlighting their potential as both standalone and synergistic agents [45]. With over 4000 years of documented human use and therapeutic versatility, cannabinoids present a promising avenue for the development of novel treatments for infections caused by *S. aureus* and GAS [46,47,48,49,50,51,52,53,54].

This systematic and critical review aims to evaluate the antibacterial efficacy of medicinal cannabis against *S. aureus* and *S. pyogenes*, including resistant strains. It will also explore potential synergistic applications of cannabinoids and examine the structure–activity relationships of active compounds to understand their therapeutic mechanisms better.

## 2. Results

**Figure 1:**
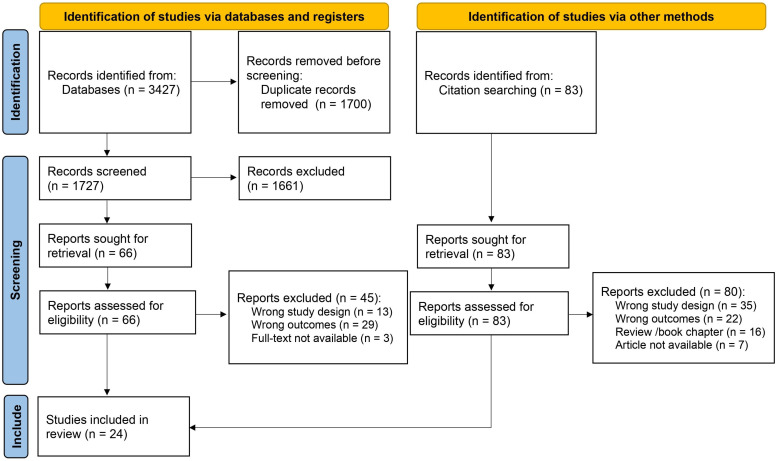
Study selection flow diagram.

Our initial search identified 3427 reports (Supplementary Material S1). Following duplicate removal (n = 1700), we screened the titles and abstracts of the remaining 1727 records, excluding 1661 reports. After a detailed full-text review of the 66 remaining studies, we found 21 that met the final inclusion criteria (Figure 1). Citation searches of these articles and relevant reviews yielded 83 reports, of which three fulfilled the inclusion criteria. In total, 24 articles were extracted. The list of abbreviations used in this review are given at the end of this article.

### 2.1. Characteristics and Quality of the Selected Studies

The studies included in our review were published between 1976 [55] and 2022 [56] and consisted of one abstract (accompanied by a poster) [57], one letter [39], and 22 peer-reviewed research publications [40,55,56,58,59,60,61,62,63,64,65,66,67,68,69,70,71,72,73,74,75,76]. All studies reported minimum inhibitory concentrations (MICs), with six studies [67,68,70,71,74,76] providing data on minimum bactericidal concentrations (MBCs). Twenty articles presented MICs and/or MBCs against *S. aureus* or methicillin-susceptible *S. aureus* (MSSA) [40,55,56,57,58,60,61,62,63,64,65,66,67,68,69,71,72,73,74,76], while eleven publications reported these outcomes against MRSA and/or vancomycin-resistant *S. aureus* (VRSA) [39,56,57,59,63,66,67,68,72,75]. Four articles detailed MICs and/or MBCs against *S. pyogenes* [40,55,56,57]. The substances investigated ranged from cannabis extracts and essential oils to isolated, semi-synthetic, or fully synthetic compounds, some of which were procured from vendors or produced via fermentation.

Risk of Bias (RoB) scores for the selected studies primarily fell into categories 1 and 3. Both Ali et al. (2012) [58] and Schuetz et al. (2021) [57] were assigned a score of 3 due to the complete absence of a methodology description for the MIC. Several other studies scored 3 because they failed to report the CBD concentrations tested against the bacteria during the MIC determination [39,60,62,68,69,71,75] or neglected to mention the inclusion of negative control [63,65]. If these essential aspects were reported, these studies would primarily be included under RoB category 1 [39,60,62,63,65,68,69,71,75]. Two studies, Turner and Elsohly (1981) [73] and van Klingeren and Ten (1976) [55], lacked tested concentrations and negative control documentation. If this information was provided, their respective RoB scores would be adjusted to categories 2 [73] and 1 [55], respectively. The omission of test concentrations and negative controls greatly influences the RoB scores, as these are mandatory reporting details according to the Toxicological Data Reliability Assessment Tool (ToxRTool 2009, European Commission’s Joint Research Centre, Ispra, Italy). The absence of these data significantly lowers the reliability score of the study.

The MIC determination in the reviewed studies employed a range of assays, including broth macrodilution, broth microdilution, agar plate dilution, and agar well diffusion assays, with broth microdilution being the most frequently utilised. The researchers either adhered to the European Committee on Antimicrobial Susceptibility Testing (EUCAST), International Standards Organisation (ISO) standards, or to guidelines from the Clinical and Laboratory Standards Institute (CLSI), previously known as the National Committee for Clinical Laboratory Standards (NCCLS). However, most researchers did not indicate whether they followed specific guidelines. The studies utilising the broth microdilution method displayed considerable variability regarding their final bacterial inoculum (ranging from 10^3^ to 1.5 × 10^8^ CFU/mL), culture medium (using cation-adjusted Mueller–Hinton broth, Mueller–Hinton broth, Brain Heart Infusion, nutrient broth, or Luria–Bertani broth), incubation time (ranging from 3 to 48 h), type of plate, and choice of solvent for stock solution preparation (with options including methanol, dimethyl sulfoxide, sterile water, ethyl acetate, phosphate-buffered saline with a pH of 7.0, Tween 80, or ethanol). Notably, some studies failed to report these specific details, especially regarding the type of 96-well plate employed. A comprehensive overview of these study characteristics, including study objectives, a detailed examination of cannabinoids, the method employed for MIC and MBC determination, study limitations, and overall study quality, is provided in Supplementary Table S1. It should be noted that information about purity is included only when specified in the original publication.

### 2.2. The Antistaphylococcal Effects of Cannabinoids

Based on the reviewed literature, CBD was the most frequently studied and potent cannabinoid against *S. aureus* [40,55,56,57,64,66]. Table 1 summarises the in vitro effects of cannabinoids on *S. aureus*. The observed MIC for CBD ranged between 0.65 and 32 mg/L against *S. aureus* [40,55,56,57,64,66].

**Figure 2:**
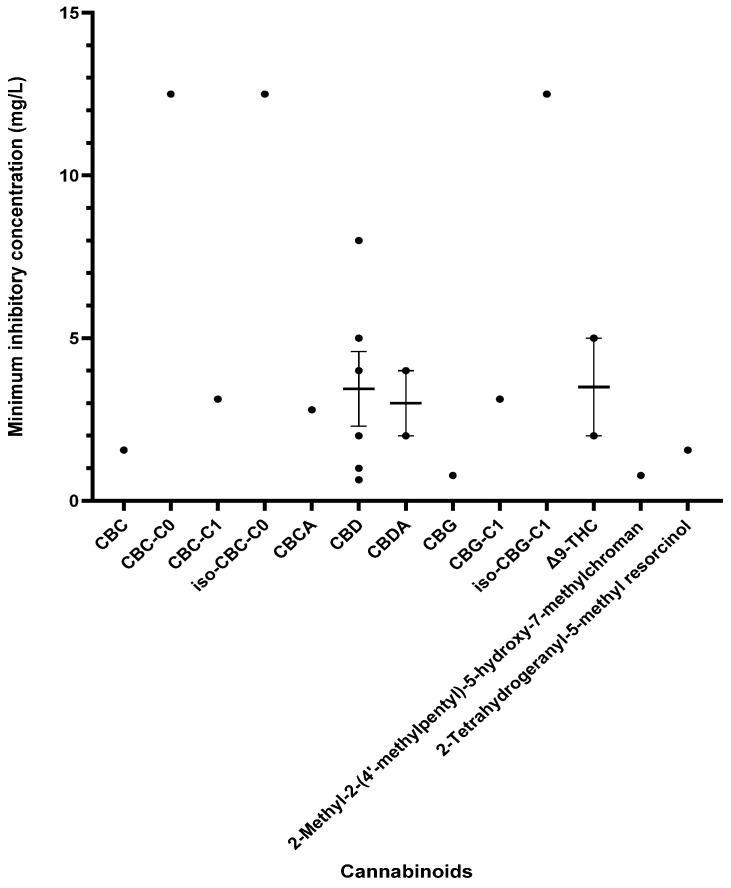
A comparison of cannabinoids demonstrating in vitro activity against *S. aureus* (MIC values < 16 mg/L) in a blood-free medium. Cannabidiol (CBD) also reported an MIC of 32 mg/L [64]. The mean and standard error of the mean (excluding outliers) are shown for compounds with multiple MIC values. Abbreviations used in this Figure are listed at the end of this article.

**Figure 3:**
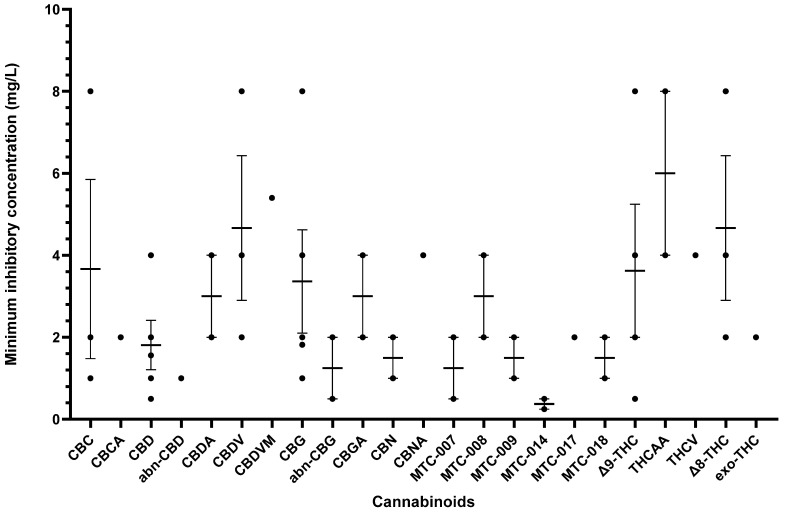
A comparison of cannabinoids with in vitro MIC values (<16 mg/L) against MRSA. Compounds such as CBDA, CBNA, and THCV with MIC values ≥ 16 mg/L were excluded from the graph. Mean MIC and standard error are shown for compounds with multiple values. Abbreviations are listed at the end of the article.

Other cannabinoids showing significant antibacterial activity included CBC, CBC-C_0_, CBC-C_1_, iso-CBC-C_0_, CBCA, CBDA, CBG, CBG-C_1_, iso-CBG-C_1_, Δ9-THC, and several other specialised derivatives [55,57,61,63,66,70,73]. The clinical potential of crucial cannabinoids with noteworthy activity (MIC < 16 mg/L) against *S. aureus* is illustrated in Figure 2. Of these, the majority showed MIC ≤ 4 mg/L (CBC, CBC-C_1_, CBCA, CBD, CBDA, CBG, CBG-C_1_, Δ9-THC, and other specialised derivatives), suggesting susceptibility of *S. aureus* to these agents [79]. The compounds with MICs of 8–16 mg/L show dose-dependent susceptibility, which can be considered when the causative bacteria is resistant to other drugs [79]. It was observed that the derivatives of parent compounds (CBC, CBG) exhibited higher MICs compared to the parent compounds. Table 2 offers an all-inclusive overview of the in vitro antibacterial capabilities of cannabinoids against MRSA and VRSA, along with the specifics of the susceptibility assays employed. The most effective cannabinoids against MRSA comprised CBC, CBCA, CBD, abn-CBD, CBDA, CBG, abn-CBG, CBGA, CBN, CBNA, Δ9-THC, THCV, exo-THC, and some synthetic derivatives as shown in Figure 3 [39,40,56,57,59,63,66,75]. Against VRSA, only CBD was evaluated, and it exhibited good activity with an MIC of 1–2 mg/L [40].

A few studies have provided data on the MBC for cannabis extracts or cannabinoids against *S. aureus* [67,68,71,74,76]. Notably, the MBC values were either equivalent to or slightly higher (by one dilution in two-fold dilution increment) than the corresponding MICs against *S. aureus* [67,68,71,76]. However, only a pair of studies reported MBC values for cannabis extracts in relation to MRSA [67,68], while one study [70] reported MBC for cannabinoids against *S. aureus*.

**Table 2:** A summary of the in vitro activity of cannabinoids (MIC and MBC data) against MRSA and VRSA.

Reference	Compound/Extract/EO	Bacteria (Type/Source)	Antimicrobial Outcome (MIC, MBC)	Reference Data
Muscara et al., 2021 [68]	0.1% acetic acid/hexane extract of dried flowering tops of *C. sativa* Chinese accession (G-309) as such and after hydrodistillation of the essential oil	MRSA (n = 19) (clinical isolates)	MBC (mg/L) = 39.06–78.13 (both extracts)	MBC (mg/L) VAN = 0.31–0.62
Sarmadyan et al., 2014 [72]	Hydro-alcoholic extract of *C. sativa*	MRSA	MIC (mg/L) = 50	
Muscara et al., 2021 [67]	0.1% acetic acid/hexane extract of dried flowering tops *C. sativa* L. var. *fibrante* as such and after hydrodistillation of the essential oil	MRSA (n = 19) (clinical isolates)	MBC (mg/L) = 4.88–78.13	MBC (mg/L) VAN = 0.32–0.64
Abichabki et al., 2022 [56]	CBD	MRSA (ATCC 43300, N315, ATCC 700698 [Mu3], ATCC 700699 [Mu50])	MIC (mg/L) = 4	PMB (MIC not given)
Wassmann et al., 2020 [75]	CBD	MRSA (USA300 FPR3757)	MIC (mg/L) = 4	-
Schuetz et al., 2021 [57]	CBD, CBG	MRSA (ATCC 33591)	MIC (mg/L) CBD = 1.56 CBG = 1.82	MIC (mg/L) DOX = 20.83
Blaskovich et al., 2021 [40]	CBD, CBDA, CBDV, 7-hydroxycannabidiol, MTC-002, MTC-005, MTC-007, MTC-008, MTC-009, MTC-011, MTC-012, MTC-013, MTC-014, MTC-017, MTC-018, 7-nor-7-carboxycannabidiol, 7-nor-7-hydroxymethyl-cannabidivarin, 7-nor-7-carboxy-cannabidivarin, Δ9-THC [80,81], THCV, THCVA, THCA-A, Δ8-THC, CBG, CBGA, CBNA	Aerobic assay MRSA (n ≥ 4) (ATCC 43300, ATCC 700699:NRS-1, ATCC 33591, clinical isolates) VRSA (n ≥ 4) (VRS1) Anaerobic assay MRSA (n ≥ 4) (ATCC 43300)	Aerobic assay MIC (mg/L) against MRSA (ATCC 43300) CBD = 1–2 CBDA = 16–32 CBDV = 2–4 7-hydroxycannabidiol = 16 MTC-002 = 16 MTC-005 = 16 MTC-007 = 0.5–2 MTC-008 = 2–4 MTC-009 = 1–2 MTC-011 ≥ 64 MTC-012 > 64 MTC-013 > 64 MTC-014 = 0.25–0.5 MTC-017 = 2 MTC-018 = 1–2 7-nor-7-carboxycannabidiol ≥ 64 7-nor-7-hydroxymethyl-cannabidivarin = 64 7-nor-7-carboxy-cannabidivarin >64 Δ9-THC = 4–8 THCV = 64 THCVA = 32–64 THCA-A = 8 Δ8-THC = 4–8 CBG = 4–8 CBGA = 2–4 CBNA = 4–16 MIC (mg/L) against MRSA (NRS1) CBD = 1–4 MIC (mg/L) against VRSA CBD = 1–2 Anaerobic assay MIC (mg/L) against MRSA CBD = 1–2	Aerobic assay MIC (mg/L) against MRSA VAN = 0.5–4 DAP = 0.5–8 TMP = 2–4 Mupirocin = 0.125–0.5 CLI ≥ 64 MIC (mg/L) against VRSA VAN > 64 DAP = 1–4 TMP >64 Mupirocin = 32–64 CLI >64 Anaerobic assay MIC (mg/L) against MRSA VAN = 05–1 ERY > 32 TET = 0.06–0.25 Mupirocin = 0.03–0.06 CLI > 32
Farha et al., 2020 [39]	CBC, CBCA, CBD, CBDA, CBDV, CBDVA, CBG, CBGA, CBL, CBN, (−)Δ8-THC, (−)Δ9-THC, exo-THC, THCAA, THCV, THCVA, (±)11-nor-9-carboxy- Δ9-THC, (±)11-hydroxy- Δ9-THC	MRSA (n = 1) (USA300)	MIC (mg/L) CBC = 8 CBCA = 2 CBD = 2 CBDA = 16 CBDV = 8 CBDVA = 32 CBG = 2 CBGA = 4 CBL > 32 CBN = 2 Δ9-THC = 2 Δ8-THC = 2 exo-THC = 2 THCAA = 4 THCV = 4 THCVA = 16 (±)11-nor-9-carboxy- Δ9-THC > 32 (±)11-hydroxy Δ9-THC > 32	
Appendino et al., 2008 [59]	Δ9-THC, 3′-bis-prenyl CBD, abn-CBD, carmagerol, CBC, CBD, CBD-di-Ac [82], CBDA [83], CBDM [84], CBD dimethyl ether [84], CBDA methyl ester [85], CBG, CBGA [86], CBGA methyl ester, CBG-di-Ac, CBG monomethyl ether, CBG dimethyl ether, CBN, phenethyl ester of CBDA, phenethyl ester of CBGA, THCAA [39]	MRSA (n = 6) (SA-1199B, RN-4220, XU212, ATCC25923, EMRSA-15, EMRSA-16)	MIC (mg/L) CBDA = 2 CBD = 0.5–1 CBC = 1–2 CBGA = 2–4 CBG = 1–2 CBGA methyl ester (tested against SA-1199B and XU212): 64 THCAA = 4–8 Δ9-THC = 0.5–2 CBN (tested against SA-1199B, RN-4220, XU212, ATCC25923, EMRSA-15) = 1 abn-CBD = 1 abn-CBG (tested against SA-1199B, RN-4220, XU212, ATCC25923, EMRSA-15) = 0.5–2 carmagerol = 16–32 CBD-di-Ac > 128 CBDM > 128 CBD dimethyl ether > 128 CBDA methyl ester > 128 phenethyl ester of CBDA > 128 CBG-di-Ac > 128 CBG monomethyl ether > 128 CBG dimethyl ether > 128 phenethyl ester of CBGA > 128 3′-bis-prenyl CBD > 128	Norfloxacin = 0.5–128 ERY = 0.25–>128 TET = 0.125–128 OXA = 0.125–>128
Galletta et al., 2020 [63]	CBCA, CBCM, CBCTFA, CBDVM, CBLM	MRSA (n = 1) (Clinical isolate)	MIC (µM) CBCA = 3.9 (1.4 mg/L) CBCTFA > 250 CBLM > 250 CBCM > 250 CBDVM = 15.6 (5.4 mg/L)	-
Martinenghi et al., 2020 [66]	CBD, CBDA	MRSA (n = 1) (USA300)	MIC (mg/L) CBD = 1 CBDA = 4	MIC (mg/L) CLI = 128 TOB = 1 MEM = 16 OFX = 64

The list of abbreviations used in this Table are given at the end of this article. Number of tested bacteria and source are given only when reported in the respective articles.

CBD exhibited concentration-dependent rapid bactericidal activity, with a bactericidal effect observed within 2–3 h at a concentration of 2 mg/L [40,66]. Similarly, cannabidiolic acid (CBDA) showed rapid bactericidal action at a concentration of 40 µM (14.3 mg/L), albeit with bacterial regrowth observed after eight hours [63]. These findings are encapsulated in Table 3 for ease of reference.

### 2.3. The Synergistic and Additive Effects of Cannabinoids

The synergistic effects of CBD and essential oils of *C. sativa* with antibacterial agents like bacitracin (BAC) and ciprofloxacin were studied against *S. aureus* and MRSA [66,69,75]. Pairing of CBD and BAC resulted in synergistic bactericidal action against MRSA (USA300) [75]. In contrast, CBD did not exhibit any synergy when combined with antibiotics like dicloxacillin, daptomycin, nisin, or tetracycline against MRSA USA300 [75], as detailed in Table 4. The synergistic analysis of cannabinoids against *S. pyogenes* was not explored in the studies examined.

### 2.4. Antibiofilm Effects

Compounds such as CBC, CBCA, CBD, CBG, CBGA, THCV, Δ8-THC, and exo-THC showed promise against *S. aureus* and/or MRSA [39,40,62,76]. Interestingly, these activities were present at concentrations equal to or slightly above the corresponding MIC values, making the clinical usefulness of cannabinoids particularly beneficial [62]. For instance, the MICs of CBC, CBCA, CBD, CBG, CBGA, Δ8-THC, exo-THC, and THCV ranged from 0.5 to 8 mg/L, whereas MBECs of respective compounds ranged from 2 to 8 mg/L. A comprehensive summary of these outcomes is provided in Table 5.

### 2.5. The Antistreptococcal Effects of Cannabinoids

**Figure 4:**
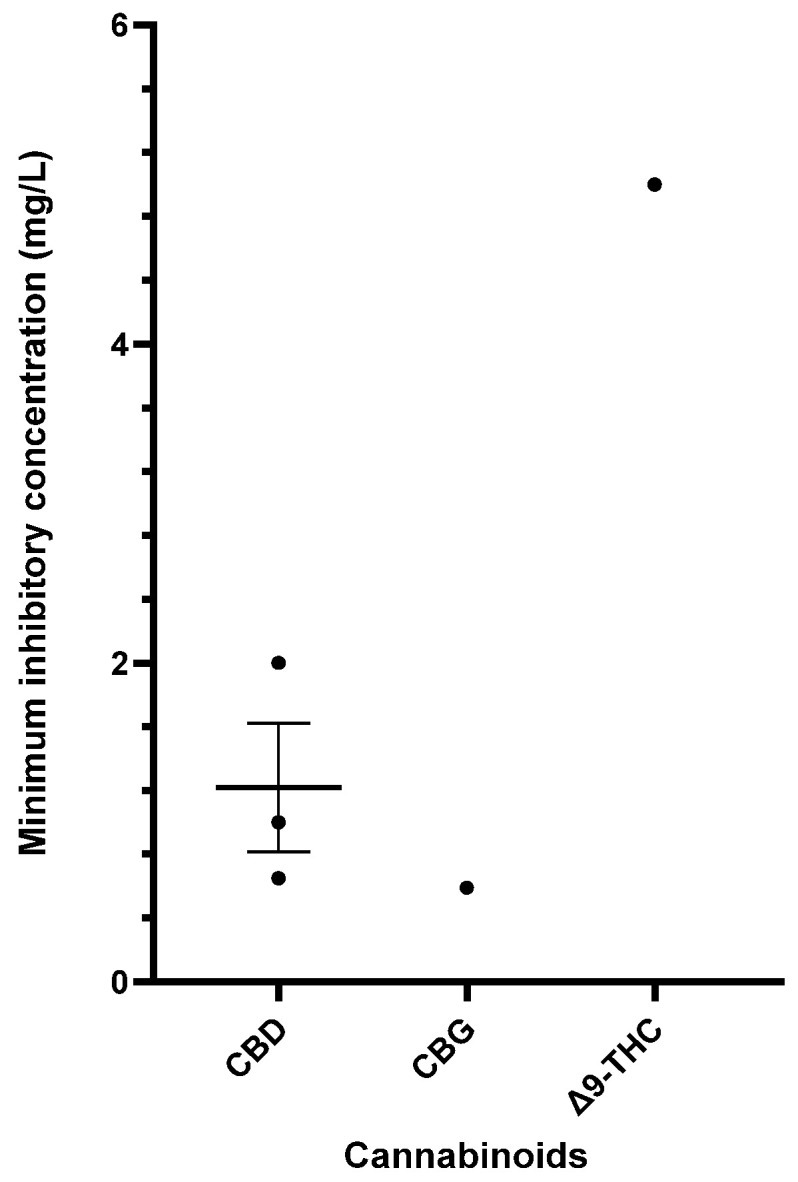
Comparison of cannabinoids demonstrating in vitro activity against *S. pyogenes* (CBD MIC: 32–50 mg/L; THC MIC: 50 mg/L). The mean and standard error are presented for compounds with multiple MIC values, excluding outliers. Abbreviations used in this Figure are provided at the end of the article.

Table 6 provides a comprehensive summary of the in vitro antibacterial effects of cannabinoids on *S. pyogenes*, detailing the MICs and MBCs and relevant specifics of the susceptibility assays. Exploration of cannabinoids against *S. pyogenes* remains limited, but CBD, CBG, and Δ9-THC have demonstrated significant antibacterial results [40,55,56,57]. Figure 4 furnishes a side-by-side comparison of different cannabinoids showing promising MIC data (<16 mg/L) against *S. pyogenes*.

**Figure 5:**
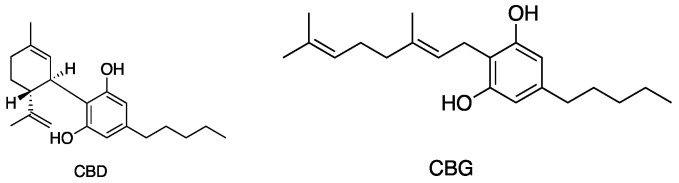
Structures of cannabinoids active against *S. aureus*, MRSA, and *S. pyogenes*; CBD is also active against VRSA. The list of abbreviations used in this Figure are given at the end of this article.

**Figure 6:**
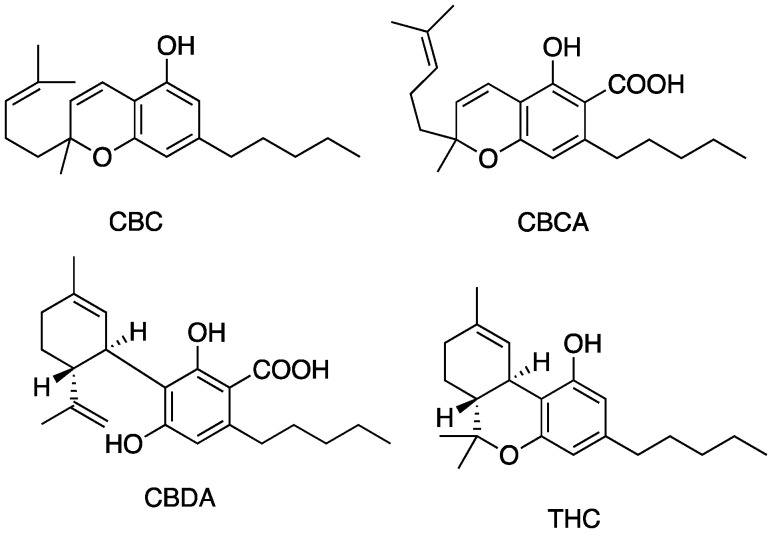
Structures of cannabinoids active against both *S. aureus* and MRSA (but not *S. pyogenes*). The list of abbreviations used in this Figure are given at the end of this article.

**Figure 7:**
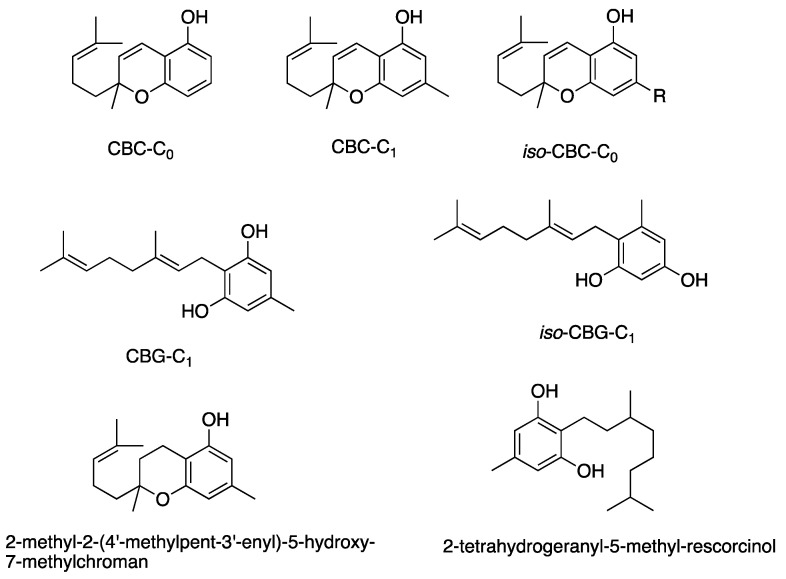
Structures of cannabinoids active against *S. aureus.* The list of abbreviations used in this Figure are given at the end of this article.

**Figure 8:**
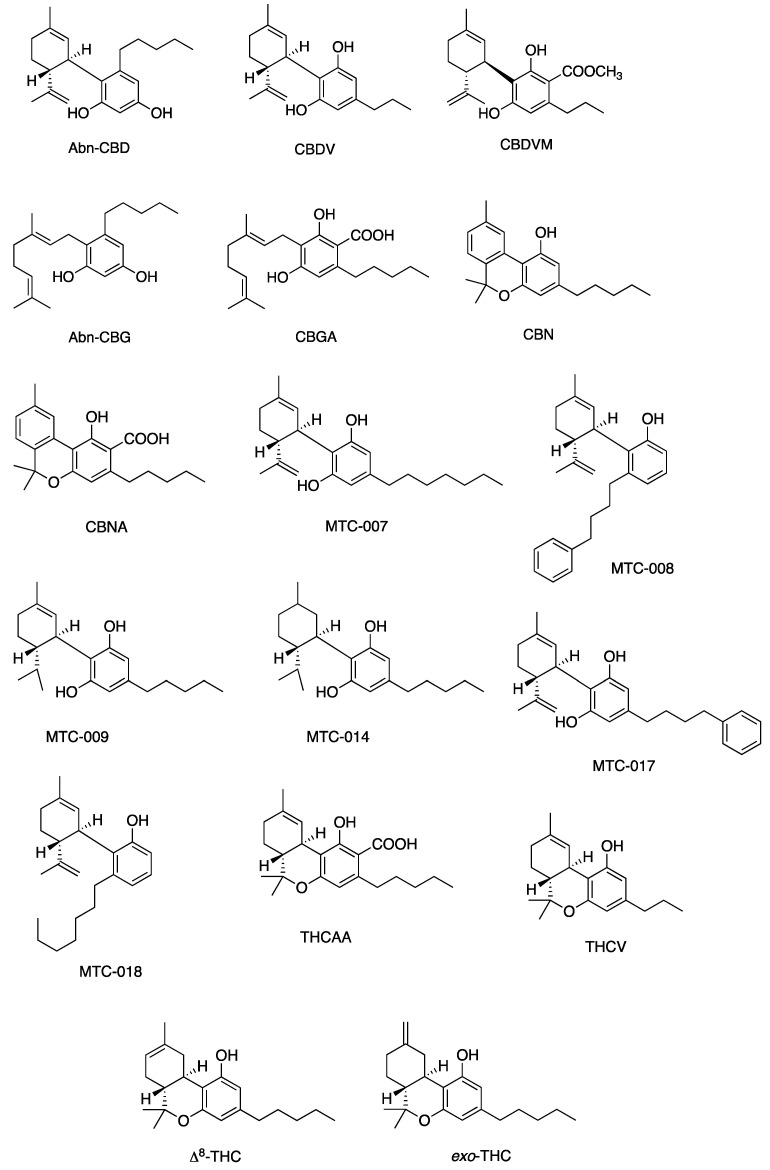
Structures of cannabinoids active against MRSA only. The list of abbreviations used in this Figure are given at the end of this article.

Figure 5, Figure 6, Figure 7 and Figure 8 describe the chemical structures of the cannabinoids most effective against distinct bacterial strains. Figure 5 includes those active against *S. aureus*, MRSA, and *S. pyogenes*; Figure 6 shows those active against both *S. aureus* and MRSA; Figure 7 highlights those effective against *S. aureus* exclusively; and Figure 8 focuses on those specific to MRSA. All depicted compounds were identified as having MIC values of less than 16 mg/L.

### 2.6. Structure–Activity Relationships in Cannabinoids and Antibacterial Implication

**Figure 9:**
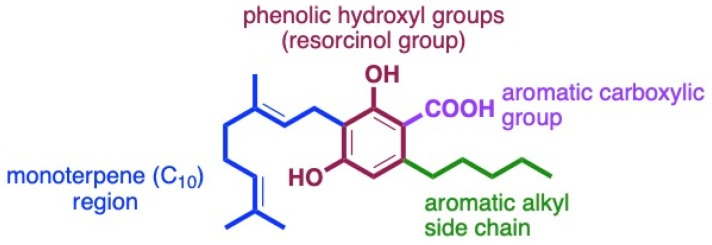
Four main sites for structurally and biologically diverse cannabinoids.

The structural features of cannabinoids can be divided into four main categories (Figure 9), each having specific structure–activity relationships (SARs):

1. Monoterpene Region (C_10_): This region is crucial for synthesising THC-type tricyclic cannabinoids through rearrangements and cyclisation.
2. Aromatic Alkyl Side Chain: Usually consisting of a 5xC chain in cannabinoids, called olivetoids, variations include 3xC varinoids (e.g., CBDV, CBDVM), 1xC orcoids (CBC-C_1_), and resorcinoids without side chains (e.g., CBC-C_0_).
3. Resorcinol Group: Comprised of two phenolic hydroxyl groups, this part can form cannabinoids like Δ9-THC through cyclisation with monoterpenes. These groups may also be subject to O-methylation and O-acetylation reactions.
4. Aromatic Carboxylic (COOH) Groups: Found in cannabinoids, these groups are essential in biosynthesis. CBGA, with its aromatic COOH group, is a precursor for other cannabinoids like Δ9-THC, CBD, CBG, and CBN, undergoing decarboxylation, rearrangements, and other modifications [35,59,78,86,88].

The study of the SARs of natural cannabinoids is well established [35,59,88]. The summarised antibacterial activity and SAR reveal essential features for antibacterial efficacy, particularly against Gram-positive pathogenic bacteria such as *S. aureus*, MRSA, and *S. pyogenes* [35,40].

The monoterpene region within cannabinoids significantly influences both biosynthesis and antibacterial effectiveness. During biosynthesis, a C_10_-monoterpene moiety is inserted at the 2-resorcinol position through the action of the enzyme aromatic prenyltransferase. This prenyl group can exist in several forms, including an acyclic structure in CBG and CBGA. Compared to its cyclic counterpart, the acyclic form reduces antibacterial activity by 2- to 4-fold. In CBD, a cyclohexene structure increases potency, but extending the ring, as in Δ9-THC, halves the activity against *S. aureus*. As in CBN, incorporating an extra aromatic ring does not alter antibacterial function. Conversely, transforming alkenes into alkanes (seen in MTC-014) enhances activity 2- to 4-fold relative to CBD. Any hydroxylation or oxidation that diminishes the lipophilicity of the prenyl group, such as in the tail of CBG or the cyclohexene ring, negates this activity, making it less effective than CBD. Therefore, maintaining the lipophilic nature of the prenyl group is vital for effective antibacterial function. The variations within the monoterpene region are pivotal, leading to nuanced differences in the efficacy of cannabinoids against bacterial strains [35,39,40,55,59,66,73,80,86].

The aromatic alkyl side chain, originating from hexanoyl-CoA, plays a crucial role in the antibacterial properties of cannabinoids, as introduced during the early stages of biosynthesis. The position of this chain—specifically at the third position (meta) to the phenolic hydroxyl group—appears vital for antibacterial activity. Its length, ranging from C-0 to C-7, produces distinct activity profiles. For example, lengthening CBD’s aromatic alkyl chain from C-5 to C-7, as in the compound MTC-007, results in similar or doubled antibacterial effectiveness. In contrast, a reduction to C-3, as in CBDV, causes a 2-fold decrease in activity. The Turner group’s study on cannabichromene (CBC) derivatives revealed a similar trend. The C-5 chain configuration exhibits the highest potency among CBC types, while deviations from this length lead to progressively reduced activity. Shortening to C-1 (CBC-C_1_) or C-0 (CBC-C_0_) reduces antimicrobial strength by 8- and 16-fold, respectively. Conversely, extending the chain up to C15, as seen in 2-methyl-2-(4′-methylpent-3′-enyl)-5-hydroxy-7-pentadec-8″-enylchromene, substantially diminishes the activity. Overall, these findings highlight the sensitivity of cannabinoids’ antibacterial performance to the aromatic alkyl side chain length [35,39,40,55,59,66,73,80,86].

The presence of an aromatic carboxylic acid group, along with two phenolic hydroxyl groups in cannabinoids, results from the polyketide pathway. The initial stages of this pathway involve the enzymatic fusion of malonyl-CoA with hexanoyl-CoA, leading to a series of reactions that create CBGA. This compound serves as a precursor for various cannabinoids. Removing the COOH group is a non-enzymatic process that can occur spontaneously. Factors such as temperature, light exposure, storage conditions, and specific cofactors can facilitate this removal. Consequently, isolating carboxylate analogues is often challenging. In examining the SAR of cannabinoids, the presence or absence of the aromatic COOH group has a profound impact. Specifically, CBDA, the carboxylated form of CBD, exhibits a 16- to 32-fold reduction in antibacterial activity compared to CBD. This decrease in activity suggests that eliminating the COOH group can increase the antibacterial potency, possibly by making the compound less hydrophilic, thereby affecting membrane permeability. Conversely, when the COOH group is esterified, as in CBG–methyl ester or CBG–ethyl phenyl ester, the antibacterial activity is eradicated (>64 mg/L) in comparison to the parent CBGA molecule (2–4 mg/L). This activity loss may stem from the increased hydrophobicity introduced by these substitutions, which further diminishes the compound’s hydrophilicity [35,39,40,55,59,66,73,80,86,88].

Phenolic Hydroxyl Groups in Cannabinoids: Derived at the onset of biosynthesis, the two phenolic hydroxyl groups are pivotal for preserving the biological functions of cannabinoids. These can be either free (as in CBD) or involved in prenyl group cyclisation (as in Δ9-THC). Regardless of being free or cyclised, these groups enhance activity. However, altering these groups through acetylation or methylation significantly impacts the antibacterial properties. Specifically, in compounds MTC-012 and MTC-013—both derived from CBD—the methylation of one or both hydroxyl groups leads to a loss in antibacterial activity. Likewise, removing one hydroxyl group in MTC-011 decreases efficacy against bacteria such as *S. aureus*. The significance of the aromatic hydroxyl groups lies in their role in stabilising hydrophilicity, thereby contributing to antibacterial properties. For instance, the interchange of the OH at C1 with the pentyl group at C3 in CBD and CBG leaves the antibacterial activity intact. The crucial requirement is the presence of these groups at specific positions (C_1_ and C_3_) on the aromatic ring. It is important to note that the resorcinol molecule lacks antibacterial activity without supporting other structural elements, such as monoterpene and aromatic alkyl groups [35,39,40,55,59,66,73,80,86].

In a broader context, the structural elements contributing to potent antibacterial activity include a decarboxylated aromatic ring with phenolic hydroxyl groups at the 1,3 positions, an aromatic alkyl chain meta to the hydroxyl groups, and a non-polar lipophilic prenyl group.

## 3. Discussion

To the best of the authors’ knowledge, this systematic and critical review represents the first comprehensive examination of the antistaphylococcal and antistreptococcal properties of cannabinoids. Various cannabinoids have demonstrated significant antibacterial efficacy against *S. aureus*, including MRSA. Although studies on the antibiotic capabilities of cannabinoids against VRSA and *S. pyogenes* are limited, initial results appear encouraging. The alignment of MBCs and MBECs with respective MICs highlights cannabinoids as a noteworthy option amidst the growing concerns over AMR.

With the escalating challenge of AMR, exploring alternative antibacterial agents has become a pressing need [89,90], and *C. sativa* emerges as a plant source of untapped potential. Cannabinoids are increasingly recognised for their therapeutic roles in pain management, acne vulgaris, dermatitis, pruritus, wound healing, and scleroderma, with recent studies pointing to potential benefits in paediatric dermatology [46,47,48,49,50,51,52,53,54,91,92,93,94].

### 3.1. Safety Profile of Cannabinoids

The safety profile of cannabinoids further underscores their viability. For instance, acute oral administration of cannabis or THC has been found to be non-lethal in dogs and monkeys, with single doses revealing no acute toxicity [95,96]. Low doses of Δ9-THC cause decreased locomotion and sedative and stimulant effects in animals [96]. Escalated CBD doses of up to 62 mg/kg were well tolerated in dogs, showing only minor, placebo-comparable side effects [97]. In rodents, single doses of oral or intraperitoneal cannabinoids (CBD and CBG at 120 mg/kg; 60 mg/kg CBDV and 30 mg/kg Δ9-THC) resulted in piloerection and drowsiness [95]. Chronic CBD administration in humans up to 1500 mg/day was well tolerated [98,99]. Short-term topical and transdermal applications in humans have shown no sensitizing or irritating effects [99,100]. Short-term oral CBD + THC administration (16 days) was well tolerated in human studies, with no notable differences in blood and liver tests and no significant dermatological side effects [101]. Topical 1% CBD cream was tested in a mouse model of autoimmune encephalomyelitis [102]. However, the authors had not reported on the presence or absence of side effects [102]. A case series revealed that topical cannabinoids (THC and/or CBD) caused no side effects in patients with chemotherapy-induced neuropathy, except for one instance where discontinuation led to transient worsening neuropathy [103]. These suggest that cannabinoids exert only minor adverse effects.

### 3.2. Pre-Clinical Effectiveness

A 5% CBD topical formulation was effective against a mouse model of *S. aureus* skin infection [40]. Topical CBD, either alone or incorporated in nanoparticles, accelerated the healing of both acute and chronic MRSA-infected wounds in a murine model [104]. Tetrahydrocannabidiol ointment reduced the bacterial load to a minimum level on day 5, comparable with that of mupirocin on dermal MRSA infections on a murine model [105]. However, systemic administration of CBD (100 mg/kg subcutaneously, 200 mg/kg intraperitoneally, or 250 mg/kg orally) was ineffective in an immunocompromised mouse model with MRSA thigh infection [40]. These findings led to phase II clinical trials investigating topical CBD for *S. aureus* nasal colonization, which is now completed [40,44,106]. The non-lethal nature and favourable safety record of cannabinoids underscore their potential suitability as promising alternatives to antibacterials [95,96,97,98,100,107]. Moreover, a recent systematic review on pre-clinical and clinical effectiveness of cannabinoids in skin wound healing and antibacterial potential summarised the potential of CBD and tetrahydrocannabidiol in reducing bacterial loads in SSTIs [45].

### 3.3. Efficacy of Cannabinoids Against S. aureus, MRSA and VRSA

MICs of cannabinoids against *S. aureus* range from 0.78 to 32 mg/L in media without blood, with most values below 4 mg/L. In comparison, existing antibiotics exhibit varying levels of activity against different *S. aureus* strains, such as fusidic acid (MICs ≤ 0.06 to ≥32 mg/L), mupirocin (0.03 to ≥1024 mg/L), retapamulin (0.03 to 128 mg/L), ozenoxacin (≤0.001 to 2 mg/L), ampicillin (0.125–32 mg/L), cefoxitin (2–64 mg/L), cefuroxime (0.094–512 mg/L), cephalexin (8–16 mg/L), chloramphenicol (0.2–256 mg/L), clindamycin (0.5–250 mg/L), gentamicin (0.016–512 mg/L), oxacillin (0.125–8 mg/L), penicillin (0.016–48 mg/L), trimethoprim–sulfamethoxazole (0.25–8 mg/L), and vancomycin (0.25–12 mg/L) [40,108,109,110,111,112,113,114,115,116,117,118,119,120,121,122,123,124]. Against MRSA, cannabinoid MICs range from 0.25 to >128 mg/L, predominantly below 4 mg/L without blood in the media [39,40,56,57,59,63,66,75]. Conventional treatments like cefazolin (MICs: 0.5–≥128 mg/L), cephalexin (0.5–128 mg/L), cephalothin (0.5–512 mg/L), ciprofloxacin (0.125–≥ 16 mg/L), dalbavancin (≤0.03–>0.25 mg/L), daptomycin, fusidic acid (0.12–1024 mg/L), mupirocin (0.125–≥1024 mg/L), linezolid (≤0.25–>8 mg/L), retapamulin (0.06–8 mg/L), ozenoxacin (≤0.001–4 mg/L), trimethoprim–sulfamethoxazole (≤0.5–≥8 mg/L), vancomycin (≤0.12–64 mg/L), and other antibiotics exhibit a broad range of efficacy [108,110,111,114,115,116,119,125,126,127,128,129,130,131,132,133,134,135,136,137,138]. Naturally occurring cannabinoids often show promising activity against MRSA strains, whereas many synthetic derivatives exhibit diminished or no activity with MICs greater than 64 mg/L [40,59]. These comparative data highlight the potential of cannabinoids as alternatives to existing antibacterials with competitive MICs. Moreover, the MBCs of specific cannabis extracts, essential oils, and CBD against *S. aureus* and MRSA align with their MICs, indicating a bactericidal effect [40,68,74,76]. Interestingly, rapid bactericidal activity within 2–3 h was observed for 2 mg/L CBD and 14.3 mg/L CBCA [40,63,66]. However, regrowth of MRSA was seen after 8 h with CBCA and certain combinations (1.5 mg/L CBD with 20 mg/L bacitracin), possibly due to cannabinoid degradation or oxidation at lower concentrations and the use of a lower concentration of the cannabinoid compared to their MBCs [63,75]. At physiological pH (~7.4), cannabinoids are protonated, preventing oxidative degradation [139,140]. In stability studies at pH 7.4 in aqueous medium at room temperature, CBD remained stable for up to 24 h, whereas the stability of CBN, CBG, and CBGA decreased at 24 h [139]. The thermal instability of CBDA allows for slow decarboxylation [66]. Moreover, CBDA presents more instability than CBD since the carboxylic acid group interacts strongly with methanol and water molecules in the solvent [66]. No regrowth was observed with higher CBD and bacitracin concentrations (2 + 16 mg/L) against MRSA [75]. Moreover, CBDA reduced the MRSA load up to 8 h at higher bacterial loads where vancomycin failed [63]. Only CBD is tested against VRSA. These findings underscore the potential of cannabinoids in *S. aureus* infections.

### 3.4. Synergistic and Antibiofilm Properties of Cannabinoids

Cannabinoids enhanced the antibacterial activity of conventional antibiotics against resistant bacteria [69,75]. For instance, CBD combined with bacitracin reduced the MIC against MRSA by 32- to 64-fold [75]. CBD’s synergy with ciprofloxacin against *S. aureus* further emphasises its potential to act as a synergistic antibacterial agent [69]. Due to CBD’s low oral bioavailability, different formulations, oil-based excipients, routes of administration, and postprandial administration could enhance the clinical utility of these combinations [141,142]. These findings suggest testing CBD with existing antibiotics to prolong their effectiveness through antibiotic adjuvants [143,144,145]. Cannabinoids are also well suited for treating bacterial skin infections and wounds due to their antibacterial, wound-healing, and skin-moisturizing properties [40,104,105,146,147,148,149,150]. Additional therapeutic attributes, such as anti-pruritic and anti-inflammatory effects, may further enhance their success in these applications [42,43].

Although, CBDA exhibited re-growth of exponentially growing MRSA after 8 h, it reduced the MRSA load to undetectable limits at 4 h with no re-growth at 24 h when cell growth was arrested at the same concentration suggesting the ability to eradicate biofilms [63]. Vancomycin was effective against exponentially growing MRSA yet failed against arrested cell growth suggesting the ineffectiveness in biofilms [63]. However, not all studies have yielded positive results. Wassmann et al. (2020) [75] reported no antibiofilm effects with a CBD/bacitracin combination against MRSA, whereas essential oils and *C. sativa* extracts showed promising antibiofilm activity [62,76]. Cannabinoids’ MBECs align with their respective MICs against MSSA and MRSA, indicating their ability to kill biofilm-forming bacteria at clinically effective concentrations [39,40,151]. This makes cannabinoids able to resolve clinically challenging infections since the formation of bacterial persisters represents a significant obstacle to successful antibiotic treatment and is often implicated in recurrent infections [39,151,152]. Certain cannabinoids, like CBCA, CBGA, CBDV, and CBC, showed concentration-dependent rapid bactericidal effects on MRSA persisters, even in the stationary phase [39,63]. However, other cannabinoids (CBDA, THCVA, CBDVA, (±)11-nor-9-carboxy- Δ9-THC, (±)11-hydroxy-delta-9-THC, CBL) and vancomycin did not exhibit bactericidal activity against MRSA persisters [39]. These findings highlight the substantial potential of cannabinoids in antibacterial treatment, providing avenues for synergistic approaches, biofilm eradication, and effective action against bacterial persisters.

### 3.5. Potential of Cannabinoids Against S. pyogenes

Few studies have investigated the MICs of cannabinoids against *S. pyogenes* [40,55,56,57]. The studied cannabinoids—CBD, CBG, and delta-9-trans-THC—showed promising activity [40,55,56,57]. Van Klingeren and Ten (1976) [55] reported lower MICs for THC and CBD in media without blood than those with horse blood. However, the recommended method includes blood in the culturing medium for *S. pyogenes* [153]. Recent studies have highlighted the efficacy of CBD and CBG against *S. pyogenes*, with MICs below 1 mg/L, underscoring their therapeutic potential [40,57]. Cannabinoids’ activity against *S. pyogenes* is comparable to established antibacterials, such as fusidic acid (MICs 1–>16 mg/L), mupirocin (0.06–6.25 mg/L), retapamulin (0.016–0.25 mg/L), ozenoxacin (≤0.004–2 mg/L), amoxicillin (≤0.063–>2 mg/L), benzylpenicillin (0.023–256 mg/L), erythromycin (≤0.063–>256 mg/L), levofloxacin (0.38–1024 mg/L), penicillin (0.004–0.25 mg/L), rifampicin (64–>1024 mg/L), tetracycline (1–≥16 mg/L), trimethoprim (8–>512 mg/L), and others [110,113,114,115,116,119,154,155,156,157,158,159,160,161,162,163]. However, further research on cannabinoids’ MBC, synergistic interactions, and antibiofilm effects against *S. pyogenes* is needed.

### 3.6. Quality of Evidence

A review of existing studies reveals that most were of high quality, with the majority receiving a score of one. However, some studies were rated lower due to missing critical information, such as negative controls or specific concentrations tested. Methodological discrepancies, such as inoculum size variations, affect MIC comparisons across studies [164]. The impact of 96-well plate types on cannabinoid MICs, with polystyrene being most appropriate for CBD and its derivatives is a noteworthy observation [40]. Culture medium also significantly affected CBD MICs; shifting from CAMHB to MH-F altered CBD MICs from 4 to 64 mg/L against *S. aureus* [56]. In addition, switching from nutrient broth to horse blood agar increased MICs for CBD and THC against *S. aureus* tenfold [55]. These variations might be due to CBD’s high protein binding (≥88% in patients with hepatic impairment, 93–99% in those with normal hepatic function), impacting testing against *S. pyogenes*, where blood is required in the culturing medium [165,166]. Adhering to established guidelines (EUCAST or CLSI) would standardise experimental parameters, such as inoculum size, incubation conditions, and culture medium, and improve the understanding the full antibacterial potential of cannabinoids.

The limitations of our review include the qualitative evaluation of the data due to methodological heterogeneity and the small number of studies for a particular cannabinoid.

In summarising the existing research, various cannabinoids—including THC derivatives, CBN-type, CBD-type, CBC-type, and CBG-type—have been demonstrated to have robust antibacterial activity against *S. aureus* and MRSA. Among these, the non-psychotropic and non-sedative CBN-type, CBD-type, CBC-type, and CBG-type cannabinoids appear particularly promising for further studies [36]. To date, CBD is the only cannabinoid that has been studied against all relevant organisms of interest, including *S. pyogenes* and VRSA. This body of evidence emphasises the need for comprehensive research to deepen our understanding and realise the full antibacterial potential of cannabinoids.

## 4. Materials and Methods

The systematic review was conducted in accordance with the Preferred Reporting Items for Systematic Review and Meta-analysis guidelines (PRISMA) 2020 [167] (Supplementary Tables S2 and S3) and was registered at OSF registries [168].

### 4.1. Eligibility Criteria

This review incorporated in vitro studies examining the antibacterial effects of extracts from *Cannabis sativa*, synthetic or isolated cannabinoids, or essential oils from *C. sativa* against *S. aureus*, including MRSA and VRSA, and *S. pyogenes*. Studies merely stating the presence or absence of antibacterial properties were not considered. Studies reporting the MIC—the least concentration of an antibacterial agent that inhibits visible bacterial growth [153]—or the MBC—the smallest concentration required to kill 99.9% of the final bacterial inoculum after 24 h incubation under standard conditions [169,170]—of any form of treatment involving *C. sativa* extracts and/or cannabinoids, individually or in combination, were eligible for inclusion. These interventions were compared with either an untreated control, standard treatment, or no treatment. Studies were excluded if they failed to report either MIC or MBC against the designated bacteria or if they examined other bacterial species.

We also evaluated studies for antibiofilm activity and the antimicrobial effect of cannabinoids when combined with other agents. The minimum biofilm eradication concentration (MBEC) is the least concentration that fully eradicates bacterial biofilm [76]. The fractional inhibitory concentration (FIC), calculated from the checkerboard assay for drug combinations, measures the MIC of a drug when combined as a ratio to its standalone MIC [171]. The interaction between two drugs (A and B) in combination is indicated by the FIC index (FICI), which is the sum of their FICs (FICI = FIC_A_ + FIC_B_) [172]. FICI interpretations are ≤0.5 for synergism, 0.5–0.75 for partial synergy, 0.76–1.0 for additive effects, 1.0–4.0 for indifference, and >4.0 for antagonism [172].

### 4.2. Search Strategy

The search was conducted from the inception of each database to 24 August 2022. The search was performed using the electronic medical databases, including Cumulative Index to Nursing and Allied Health Literature (CINAHL), Cochrane Library, MEDLINE, Scopus, Web of Science, and Latin America and Caribbean Health Sciences Literature (LILACS). The comprehensive search strategy is given in Supplementary Material S1. Reference lists of all included studies and existing review articles were examined to identify additional studies. The search had no restrictions concerning the publication date, but non-English studies were not considered for data extraction. The following search query was adapted for each information source: (cannabis OR cannabinoid*) AND antibacterial*.

### 4.3. Study Selection

All articles identified during the search were transferred into EndNote 20.4.1 (Clarivate Analytics, Philadelphia, PA, USA) and then into Covidence systematic review software (2024 Covidence, Veritas Health Innovation, Melbourne, Australia). Two independent reviewers (MA, DN) carried out the study selection, data extraction, and quality assessment. Disagreements at each stage of the study selection and data extraction processes were resolved via consultation with a third reviewer (JT). The search outcome and the inclusion process are comprehensively reported in this systematic review and visualised via a PRISMA-2020 flow diagram [167]. The heterogeneity in the study methodologies precluded a meta-analysis. Rejected articles are listed in Supplementary Material S2.

### 4.4. Risk of Bias Assessment

The ToxRTool was employed to evaluate the risk of bias of in vitro studies [173]. All studies were included irrespective of their risk of bias. A reliability category 1 implies studies are reliable without restrictions, category 2 implies studies are reliable with restrictions, and category 3 indicates studies are not reliable. In contrast, category 4 suggests the score cannot be assigned.

## 5. Conclusions and Future Directions

This review evaluates the in vitro antibacterial activity of medicinal cannabis, focusing on cannabinoids and *C. sativa* extracts against *S. aureus*, MRSA, VRSA, and *S. pyogenes*. Among these, CBD exhibited marked antibacterial effects, with MICs ranging from 0.65 to 32 mg/L for *S. aureus*, 0.5–4 mg/L for MRSA, 1–2 mg/L for VRSA, and 0.6–50 mg/L for *S. pyogenes*. MBCs of CBD aligned with MIC against MRSA, suggesting the bactericidal activity at therapeutic concentrations. Cannabinoids, such as CBC, CBCA, CBD, CBG, CBGA, THCV, Δ8-THC and exo-THC, demonstrated promising antibiofilm activity against MRSA, with MBECs closely aligned to their MICs. CBD combined with bacitracin reduced the MIC for MRSA by up to 64-fold. These promising results highlight the potential of cannabinoids in overcoming bacterial resistance mechanisms.

The structure–activity relationship of cannabinoids is central to their antibacterial efficacy. The monoterpene region influences potency, as cyclic forms such as CBD are more effective than acyclic forms like CBGA. The aromatic alkyl side chain length and decarboxylation of the aromatic COOH groups enhance antibacterial properties, highlighting that maintaining the lipophilic prenyl and phenolic hydroxyl groups is essential for activity against Gram-positive bacteria.

Despite these promising results, cannabinoid research remains in its early stages, with only one clinical study on antibacterial effects to date. Further research should explore the additive and synergistic potential of cannabinoids with conventional antibiotics and other antimicrobial agents, optimise their structure–activity relationships, refine cannabinoid formulations, and enhance delivery systems to harness their therapeutic potential in clinical settings fully.

In conclusion, cannabinoids such as CBD, CBG, and Δ9-THC offer significant promise as alternatives or adjuncts to traditional antibiotics, particularly for targeting *S. aureus*, MRSA, and *S. pyogenes*. Their favourable safety profile positions them as potential candidates for antibacterial therapies, though rigorous clinical trials, standardised testing, and long-term safety studies are crucial to fully unlock their potential in combating AMR.

## Figures and Tables

**Table 1:** A summary of the in vitro activity of cannabinoids (MIC and MBC data) against *Staphylococcus aureus*.

Reference	Compound/Extract/Essential Oil	Bacteria (Type/Source)	Antimicrobial Outcome (MIC, MBC)	Reference Data
Ali et al., 2012 [58]	Seeds and the whole plant of *C. sativa* were sequentially extracted into petroleum ether and MeOH. MeOH extract was used to test MIC.	*S. aureus* (ATCC 25923)	MIC (mg/L) Seeds = 25 Whole plant = 50	-
Vu et al., 2016 [74]	MeOH extract of leaves and branches of *C. sativa*	*S. aureus* (ATCC 6538)	MIC (mg/L) = 2000 MBC (mg/L) > 2000	MIC (mg/L) Streptomycin sulfate = 5 CHL = 5 MBC (mg/L) Streptomycin sulfate = 10 CHL = 5
Sarmadyan et al., 2014 [72]	Hydro-alcoholic extract of *C. sativa*	*S. aureus* (ATCC 25923)	MIC (mg/L) = 25	-
Frassinetti et al., 2020 [62]	80% EtOH extract of *C. sativa* L. cultivar Futura 75 seeds	*S. aureus* (ATCC 25923)	MIC (mg/L) = 1000	GEN (MIC was not given) VAN (MIC was not given)
Muscara et al., 2021 [68]	0.1% acetic acid/hexane extract of dried flowering tops of *C. sativa* Chinese accession (G-309) as such and after hydrodistillation of the essential oil	*S. aureus* (n = 1) (ATCC 6538)	MIC (mg/L) = 39.06 (both extracts) MBC (mg/L) = 39.06–78.13 (both extracts)	MBC (mg/L) VAN = 0.31–0.62
Muscara et al., 2021 [67]	0.1% acetic acid/hexane extract of dried flowering tops *C. sativa* L. var. *fibrante* as such and after hydrodistillation of the essential oil	*S. aureus* (n = 1) (ATCC 6538)	MIC (mg/L) = 4.88 (both extracts) MBC (mg/L) Original extract = 4.88 After hydrodistillation of essential oil = 19.53	MBC (mg/L) VAN = 0.32–0.64
Kaur et al., 2015 [65]	Separate extractions of EtOH, MeOH, acetone, and water of *C. sativa* leaves	*S. aureus* (n = 1) (MTCC 96)	MIC (mg/L) MeOH extract = 1560 EtOH extract = 6250 Acetone extract = 6250 Water extract = 2500	-
Chauhan et al., 2017 [60]	MeOH extract of *C. sativa* leaves	*S. aureus* (n = 1) (MTCC-737)	MIC (mg/L) = 219	CIP (MIC was not given)
Pellegrini et al., 2021 [71]	EO of *C. sativa* ‘Futura 75’ inflorescence	*S. aureus* (n = 3) (St 32, St 47 and St 39)	MIC (mg/L) = 1.25–5 MBC (mg/L) = 1.25–5	-
Zengin et al., 2018 [76]	EO of *C. sativa* L. Futura 75 cultivar (aerial parts consisting of leaves, inflorescences, and thinner residues of stem)	*S. aureus* (n = 4) (ATCC 29213; clinical strains: *S. aureus* 101 TV, *S. aureus* 104, and *S. aureus* 105)	MIC (mg/L) = 8000 MBC (mg/L) = 16,000	-
Nafis et al., 2019 [69]	EO from the aerial parts of *C. sativa*	*S. aureus* (n = 1) (CCMM B3)	MIC (mg/L) = 4700	MIC (mg/L) CIP = 31
Iseppi et al., 2019 [64]	CBD; EO of *C. sativa* (from different fibre-type varieties, from inflorescences or the whole plants)	*S. aureus* (ATCC 6538, 18As, 386)	MIC (mg/L) EOs = 2–32 CBD = 8–32	MIC (mg/L) CIP = 0.5–16
Abichabki et al., 2022 [56]	CBD	*S. aureus,* (ATCC 29213, ATCC BAA-976, ATCC BAA-977)	MIC (mg/L) in CAMHB = 4 MIC (mg/L) in MH-F = 64	PMB (MIC was not given)
Blaskovich et al., 2021 [40]	CBD	MSSA, (n ≥ 4) (ATCC 25923, ATCC 29213)	MIC (mg/L) = 1–2	MIC (mg/L) VAN = 1–2 DAP = 1–2 TMP = 2–4 Mupirocin = 0.25–0.5 CLI = 0.06–0.25
Nigro et al., 2022 [70]	CBDA	*S. aureus* (n = 1) (ATCC 6538)	MIC (mg/L) = 4 MBC (mg/L) = 16	-
Martinenghi et al., 2020 [66]	CBD, CBDA	*S. aureus* (n = 1) (ATCC 25923)	MIC (mg/L) CBD = 1 CBDA = 2	MIC (mg/L) CLI > 128, TOB = 0.25, MEM = 0.06, OFX = 0.5
Schuetz et al., 2021 [57]	CBD, CBG	*S. aureus* (ATCC 29213)	MIC (mg/L) CBD = 0.65 CBG = 0.78	MIC (mg/L) DOX = 0.2
van Klingeren and Ten 1976 [55]	CBD, trans-Δ9-THC	*S. aureus* (n = 4) (ATCC 6538)	MIC (mg/L) in nutrient broth CBD = 1–5 THC = 2–5 MIC (mg/L) in horse blood agar CBD = 20–50 Δ9-THC = 20–50	-
Turner and Elsohly 1981 [73]	CBC, CBC-C_0_, CBC-C_1_, iso-CBC-C_0_	*S. aureus* (ATCC 6538)	MIC (mg/L) CBC = 1.56 CBC-C_0_ = 12.5 CBC-C_1_ = 3.12 iso-CBC-C_0_ = 12.5	MIC (mg/L) Streptomycin sulfate = 3.12–6.25
Eisohly et al., 1982 [61]	CBC [77], CBC-C_0_ [73,78], CBC-C_1_ [73,78], iso-CBC-C_0_ [73]_,_ 2-Methyl-2-(4′-methyIpentyl)-5-hydroxy-7-methylchroman, CBG-C_1,_ 2-Tetrahydrogeranyl-5-methyl Resorcinol, 2-Methyl-2-(4′-methylpent-3′-enyl)-5-hydroxy-7-pentadec-8″-enylchromene, 2-Methyl-2-(4′-methylpent-3′-enyl)–5,7-dihydroxychromene	*S. aureus* (ATCC 6538)	MIC (mg/L) CBC = 1.56 CBC-C_0_ = 12.5 CBC-C_1_ = 3.12 iso-CBC-C_0_ = 12.5 2-Methyl-2-(4′-methyIpentyl)-5-hydroxy-7-methylchroman = 0.78 CBG-C_1_ = 3.12 iso-CBG-C_1_ = 12.5 2-Tetrahydrogeranyl-5-methyl resorcinol = 1.56 2-Methyl-2-(4′-methylpent-3′-enyl)-5-hydroxy-7-pentadec-8″-enylchromene = 50 2-Methyl-2-(4′-methylpent-3′-enyl)–5,7-dihydroxychromene = 50	MIC (mg/L) Streptomycin sulfate = 6.25
Galletta et al., 2020 [63]	CBCA, CBCM, CBCTFA, CBLM, CBDVM	MSSA (34397, Clinical isolate)	MIC (µM) CBCA = 7.8 (2.8 mg/L) CBCM > 250 CBCTFA > 250 CBDVM > 250 CBLM > 250	-

The list of abbreviations used in this Table are given at the end of this article. Number of tested bacteria and source are given only when reported in the respective articles.

**Table 3:** Time-kill test results.

Reference	Compound	Organism	Time-Kill Data
Blaskovich et al., 2021 [40]	CBD	MRSA (n = 4) (ATCC 43300)	CBD exhibited rapid bactericidal activity (<3 h) with an MBC of 2 mg/L.
Martinenghi et al., 2020 [66]	CBD	MRSA (n = 1) (USA300)	CBD exhibited concentration-dependent rapid bactericidal activity, [8 mg/L (8 × MIC) was bactericidal in 30 min, 4 mg/L was bactericidal in 1 h, and 2 mg/L was bactericidal in 2 h].
Wassmann et al., 2020 [75]	CBD and BAC	MRSA (USA300 FPR3757)	The 1.5 mg/L CBD and 20 mg/L BAC were not bactericidal or bacteriostatic in the time-kill assay against MRSA (USA300 FPR3757) up to 12 h. Combination of CBD and BAC were bactericidal. But after 8 h, the combination showed re-initiation of growth, which might be a result of degradation or oxidation of CBD. The 2 mg/L CBD and 16 mg/L BAC were synergistically bacteriostatic in the time-kill assay against MRSA (USA300 FPR3757) up to 24 h.
Galletta et al., 2020 [63]	CBCA	MRSA (incubated at 5% CO_2_)	The 40 µM (14.3 mg/L) CBCA exhibited rapid bactericidal activity by reducing the number of viable bacteria to undetectable levels at 2 h post treatment. After 8 h, the bacteria showed re-growth and showed > 5-log increase at 24 h. When the growth MRSA was arrested with carbonyl cyanide m-chlorophenylhydrazone, 40 µM CBCA exhibited rapid bactericidal activity by reducing the bacterial load to undetectable levels at 4 h post treatment. Also, no re-growth was observed.

The list of abbreviations used in this Table are given at the end of this article.

**Table 4:** In vitro interactions of cannabinoids in combination with other compounds or other cannabinoids against *S. aureus* and MRSA.

Reference	Studied Agents	Other Compounds/Drugs/Formulations	Tested Combinations	Bacteria	Study Method (Medium)	Study Outcomes
Wassmann et al., 2020 [75]	CBD	BAC	½ MIC of CBD	MRSA (USA300 FPR 3757)	Not specified (FICI calculated)	FICI = 0.5 (partial synergy)
Nafis et al., 2019 [69]	EO from the aerial parts of *C. sativa*	CIP		*S. aureus* (CCMM B3)	checkerboard assay	FICI = 0.375 (synergy)
Martinenghi et al., 2020 [66]	CBD	CLI, OFX, MEM, TOB, TEC, MET, VAN	CBD: 16 mg/L to 0.25 mg/L Antibiotics: 128 mg/L to 0.5 mg/L	MRSA (USA300)	checkerboard assay (MHB)	FICI for CBD + CLI = 1.03 CBD + MEM = 1.13 CBD + MET = 1.25 CBD + OFX = 1.50 CBD + TEC = 1.13 CBD + TOB = 1.50 CBD + VAN = 1.13 (All were indifferent)

The ∑FICI values were interpreted as follows: ≤0.5 = synergistic; 0.5–0.75 = partial synergy; 0.76–1.0 = additive; >1.0–4.0 = indifferent (noninteractive); >4.0 = antagonistic [87]. The studies did not report the number of tested isolates. The list of abbreviations used in this Table are given at the end of this article.

**Table 5:** Antibiofilm effects.

Reference	Studied Agents	Bacteria	Method (Medium)	Activity
Wassmann et al., 2020 [75]	CBD CBD + BAC	MRSA USA300 (n = 1)	Static biofilm on silicone discs in 24-well plate	No effects on biofilm formation or degradation by CBD (1 mg/L) or by combination of CBD/BAC (1 mg/L/16 mg/L)
Blaskovich et al., 2021 [40]	CBD	*S. aureus* ATCC 25923 (MSSA) (n = 4) *S. aureus* ATCC 43300 (MRSA) (n = 4)	96-well polystyrene plates (TSB with 3% glucose)	MBEC (mg/L) MSSA = 1–2 * MRSA = 2–4 *
Zengin et al., 2018 [76]	EO of *C. sativa* L. Futura 75 cultivar (aerial parts consisting of leaves, inflorescences, and thinner residues of stem)	*S. aureus* (n = 4) (ATCC 29213, 101, 104, 105)	96-well polystyrene plates (TSB + 1% sucrose)	MBEC (mg/L) = 16,000–24,000 *
Frassinetti et al., 2020 [62]	80% EtOH extract of *C. sativa* L. cultivar Futura 75 seeds	*S. aureus* ATCC 35556 (n = 1)	96-well polystyrene plates (TSB + 1% sucrose) And biofilm on round cover glass slides placed in 24-well polystyrene plate	Biofilm formation was reduced by *C. sativa* seed extract at 0.1 mg/mL and 0.25 mg/mL. Biofilm formation was totally blocked by *C. sativa* seed extract at 0.5 mg/mL and 1 mg/mL with 80% inhibition rate. Inhibition pattern of biofilm formation by vancomycin (positive control) was similar to that of *C. sativa* seed extract.
Farha et al., 2020 [39]	CBC, CBCA, CBD, CBDA, CBDV, CBDVA, CBG, CBGA, CBL, CBN, (−)-Δ8-THC, (−)-Δ9-THC, exo-THC, THCAA, THCV, THCVA, (±)11-nor-9-carboxy-delta-9-THC, (±)11-hydroxy-delta-9-THC	MRSA USA300 (n = 1)	static abiotic solid-surface assays (polystyrene 96-well plates in (TSB) with 1% glucose)	Minimal biofilm eradication concentration (mg/L) CBC = 8 * CBCA = 4 * CBD = 4 * CBDA > 8 CBDV > 8 CBDVA > 8 CBG = 4 * CBGA = 4 * CBL > 8 CBN > 8 Δ9-THC > 8 Δ8-THC = 2 Exo-THC = 2 Δ9-THCA-A > 8 THCV = 8 * THCVA > 8 +/−11-nor-9-carboxy- Δ9-THC > 8 +/−11-hydroxy-Δ9-THC > 8

* MBEC is equal or slightly higher (one or two dilutions) than MIC. The list of abbreviations used in this Table are given at the end of this article.

**Table 6:** A summary of the in vitro activity of cannabinoids (MIC and MBC data) against *Streptococcus pyogenes*.

Reference	Compound/Extract/EO	Bacteria (Type/Source)	Antimicrobial Outcome (MIC, MBC)	Reference Data
Abichabki et al., 2022 [56]	CBD	*S. pyogenes* (n = 1) (ATCC 12344)	MIC (mg/L) = 32	PMB (MIC not given)
Schuetz et al., 2021 [57]	CBD, CBG	*S. pyogenes* (ATCC 19615)	MIC (mg/L) CBD = 0.65 CBG = 0.59	MIC (mg/L) DOX = 0.11
Blaskovich et al., 2021 [40]	CBD	*S. pyogenes* (n ≥ 4) (ATCC 12344, ATCC 49399, ATCC 19615, ATCC BAA-1412, ATCC BAA-1414, MMX 3820 ERY)	MIC (mg/L) Against ATCC 12344 = 1 Other *S. pyogenes* = 8–32	MIC (mg/L) VAN > 64 DAP = 1–4 TMP = >64 Mupirocin = 32–64 CLI = >64
van Klingeren and Ten 1976 [55]	trans Δ9-THC, CBD	*S. pyogenes* (n = 1)	MIC (mg/L) (nutrient broth) Δ9-THC = 5 CBD = 2 MIC (mg/L) (horse blood agar) Δ9-THC = 50 CBD = 50	-

The list of abbreviations used in this Table are given at the end of this article. Number of tested bacteria and source are given only when reported in the respective articles.

## Data Availability

Data are contained within the article and Supplementary Materials.

## Supplementary Materials

The following supporting information can be downloaded at: https://www.mdpi.com/article/10.3390/antibiotics13111023/s1, Supplementary material S1: Search strategies; Supplementary material S2: Details of rejected articles; Supplementary Table S1: Descriptive characteristics of the included studies; Supplementary Table S2: PRISMA 2020 checklist; Supplementary Table S3: PRISMA 2020 for abstracts checklist.

## Abbreviations

(−)-Δ8-THC, delta-8-tetrahydrocannabinol; (±)-CBCA, cannabichromenic acid; (±)-CBCM, cannabichromene methyl ester; (±)-CBCTFA, cannabichromene methyl ester trifluoroacetate; (±)-CBLM, cannabicyclol methyl ester; Δ8-THC, delta-8-tetrahydrocannabinol; Δ9-THC, delta-9-tetrahydrocannabinol, (−)-Δ9-tetrahydrocannabinol; abn-CBD, abnormal-cannabidiol; abn-CBG, abnormal-cannabigerol; AMR, Antimicrobial resistance; ATCC, American type culture collection; BAC, Bacitracin; BHI, Brain Heart Infusion; *C. sativa*, *Cannabis sativa;* CAMHB, Cation-Adjusted Mueller–Hinton Broth; CBC, Cannabichromene; CBC-C_0_, Cannabichromene-C_0_; CBC-C_1_, Cannabichromene-C_1_; CBCA, cannabichromenic acid; CBCM, cannabichromene methyl ester; CBCTFA, cannabichromene methyl ester trifluoroacetate; CBD, Cannabidiol; CBD-di-Ac, cannabidiol-diacetyl; CBDA, Cannabidiolic Acid; CBDM, cannabidiol monomethyl ether; CBDV, Cannabidivarin; CBDVA, cannabidivarinic acid; CBDVM, cannabidivarin methyl ester; CBG, Cannabigerol; CBG-C_1_, cannabigerol-C_1_; CBG-di-Ac, cannabigerol-diacetyl; CBGA, cannabigerolic acid; CBL, Cannabicyclol; CBLM, cannabicyclol methyl ester; CBN, Cannabinol; CBNA, cannabinolic acid A; CFU, colony forming units; CHL, Chloramphenicol; CINAHL, Cumulative Index to Nursing and Allied Health Literature; CIP, Ciprofloxacin; CLI, Clindamycin; CLSI, Clinical and Laboratory Standards Institute; CO_2_, carbon dioxide; COVID-19, coronavirus disease; DAP, Daptomycin; DMSO, dimethyl sulfoxide; DOX, Doxycycline; ED, emergency department; EO, essential oil; ERY, Erythromycin; EtOAc, ethyl acetate; EtOH, Ethanol; EUCAST, European Committee on Antimicrobial Susceptibility Testing; exo-THC, exo-tetrahydrocannabinol; FIC, fractional inhibitory concentration; FICI, fractional inhibitory concentration index; GEN, Gentamycin; H_2_, Hydrogen; ICU, intensive care unit; ISO, International Standards Organisation; iso-CBC, Isocannabichromene; iso-CBC-C_0_, isocannabichromene-C_0_; iso-CBG-C_1_, isocannabigerol-C_1_; LB, Luria–Bertani; LHB, lysed horse blood; LILACS, Latin America and Caribbean Health Sciences Literature; NCCLS, National Committee for Clinical Laboratory Standards; MBC, Minimum Bactericidal Concentration; MBEC, minimum biofilm eradication concentration; MEM, Meropenem; MeOH, Methanol; MET, Methicillin; MH, Mueller–Hinton; MH-F, Cation-Adjusted Mueller–Hinton II Broth (CAMHB) supplemented with defbrinated horse blood and β-NAD; MHA, Mueller–Hinton Agar; MHB, Mueller–Hinton Broth; MIC, Minimum Inhibitory Concentration; MRSA, methicillin-resistant *Staphylococcus aureus*; MSSA, methicillin-susceptible *Staphylococcus aureus*; MTC, name given by authors to synthetic cannabinoids; MTCC, microbial type culture collection; n, number, NA, nutrient agar; NB, nutrient broth; NCCLS, national committee for Clinical Laboratory Standards; OFX, Ofloxacin; PMB, Polymyxin B; RoB, Risk of Bias; rpm, rounds per minute; *S. aureus*, *Staphylococcus aureus*; *S. pyogenes*, *Streptococcus pyogenes*; SSTIs, Skin and soft tissue infections; TEC, Teicoplanin; TET, Tetracycline; THCA-A, THCAA, Delta-9-tetrahydrocannabinolic acid A; THCV, Δ9-tetrahydrocannabivarin; THCVA, tetrahydrocannabivarinic acid; TMP, Trimethoprim; TOB, Tobramycin; ToxRTool, Toxicological data reliability assessment tool; TSA, Tryptone Soy agar; TSB, Tryptone Soy broth; UK, United Kingdom; US, United States; VAN, Vancomycin; VRSA, vancomycin-resistant *Staphylococcus aureus*.
