# Supplementary material for: Cannabinoids as Antibacterial Agents: A Systematic and Critical Review of In Vitro Efficacy Against Streptococcus and Staphylococcus
Source: Antibiotics (Basel). 2024 Oct 30;13(11):1023. doi: 10.3390/antibiotics13111023 (PMC11591022; doi:10.3390/antibiotics13111023)
Supplement: Supplementary file 1 [file antibiotics-13-01023-s001.zip › antibiotics-3239722-supplementary.pdf]

Supplementary Materials S1. Search strategy

CINAHL plus with full text EBSCOhost

24.08.2022

No	Search	No of articles
#1	TI (cannabis OR hemp OR marijuana OR phytocannabinoid* OR cannabinoid* OR cannabichromene* OR cannabicyclol* OR cannabidiol* OR cbd OR cannabielsoin* OR cannabigerol* OR cannabinodiol* OR cannabinol* OR cannabitriol* OR tetrahydrocannabi* OR thc OR dronabinol) OR AB (cannabis OR hemp OR marijuana OR phytocannabinoid* OR cannabinoid* OR cannabichromene* OR cannabicyclol* OR cannabidiol* OR cbd OR cannabielsoin* OR cannabigerol* OR cannabinodiol* OR cannabinol* OR cannabitriol* OR tetrahydrocannabi* OR thc OR dronabinol)	23,125
#2	(MM "Cannabis+") OR (MM "Cannabinoids+") OR (MM "Cannabidiol") OR (MM "Hemp")	7,380
#3	(MM "Medical Marijuana")	1,648
#4	#1 OR #2 OR #3	23,850
#5	TI (Antibacterial* OR anti-bacterial* OR “anti bacterial*” OR antimicrobial* OR anti-microbial* OR “anti microbial*” OR “group A streptococcus” OR “streptococcus pyogenes” OR “s. pyogenes” OR “anti streptococc*” OR antistreptococc* OR anti-streptococc* OR “Staphylococcus aureus” OR “s. aureus” OR “staph aureus” OR “anti staphylococc*” OR antistaphylococc* OR anti-staphylococc* OR MRSA OR anti-MRSA OR VRSA OR anti-VRS) OR AB (Antibacterial* OR anti-bacterial* OR “anti bacterial*” OR antimicrobial* OR anti-microbial* OR “anti microbial*” OR “group A streptococcus” OR “streptococcus pyogenes” OR “s. pyogenes” OR “anti streptococc*” OR antistreptococc* OR anti-streptococc* OR “Staphylococcus aureus” OR “s. aureus” OR “staph aureus” OR “anti staphylococc*” OR antistaphylococc* OR anti-staphylococc* OR MRSA OR anti-MRSA OR VRSA OR anti-VRS)	45,537
#6	(MM "Staphylococcus Aureus+") OR (MM "Methicillin-Resistant Staphylococcus Aureus") OR (MM "Vancomycin-Resistant Staphylococcus Aureus")	6,681
#7	#5 OR #6	46,239
#8	#4 AND #7	25

Tl: title; MH: exact subject heading; MM: exact major subject heading; AB: abstract.

Cochrane library

24.08.2022

No	Search	No of articles
#1	(cannabis OR hemp OR marijuana OR phytocannabinoid* OR cannabinoid* OR cannabichromene* OR cannabicyclol* OR cannabidiol* OR cbd OR cannabielsoin* OR cannabigerol* OR cannabinodiol* OR cannabinol* OR cannabitriol* OR tetrahydrocannabi* OR thc OR dronabinol)ti,ab,kw	5,774
#2	MeSH descriptor: [Cannabis] explode all trees	381
#3	MeSH descriptor: [Medical Marijuana] explode all trees	26
#4	MeSH descriptor: [Cannabinoids] explode all trees	992
#5	#1 OR #2 OR #3 OR #4	5,774
#6	(Antibacterial* OR anti-bacterial* OR anti NEXT bacterial* OR antimicrobial* OR anti-microbial* OR anti NEXT microbial* OR group NEXT A NEXT streptococcus OR streptococcus NEXT pyogenes OR s. NEXT pyogenes OR anti NEXT streptococc* OR antistreptococc* OR anti-streptococc* OR Staphylococcus NEXT aureus OR s. NEXT aureus OR staph NEXT aureus OR anti NEXT staphylococc* OR antistaphylococc* OR anti-staphylococc* OR MRSA OR anti-MRSA OR VRSA OR anti-VRSA))ti,ab,kw	24,134
#7	MeSH descriptor: [Streptococcus pyogenes] explode all trees	275
#8	MeSH descriptor: [Staphylococcus aureus] explode all trees	870
#9	#6 OR #7 OR #8	24,134
#10	#5 AND #9	8<br>All were trials

ti,ab,kw: title, abstract and key words

Medline via EBSCOhost

24.08.2022 1.24pm

No	Search	No of articles
#1	TI (cannabis OR hemp OR marijuana OR phytocannabinoid* OR cannabinoid* OR cannabichromene* OR cannabicyclol* OR cannabidiol* OR cbd OR cannabielsoin* OR cannabigerol* OR cannabinodiol* OR cannabinol* OR cannabitriol* OR tetrahydrocannabi* OR thc OR dronabinol) OR AB (cannabis OR hemp OR marijuana OR phytocannabinoid* OR cannabinoid* OR cannabichromene* OR cannabicyclol* OR cannabidiol* OR cbd OR cannabielsoin* OR cannabigerol* OR cannabinodiol* OR cannabinol* OR cannabitriol* OR tetrahydrocannabi* OR thc OR dronabinol)	62,947
#2	(MM "Cannabis") OR (MM "Medical Marijuana")	10,739
#3	(MM "Cannabinoids+") OR (MM "Cannabidiol") OR (MM "Dronabinol") OR (MM "Cannabinol")	12,927
#4	#1 OR #2 OR #3	65,163
#5	TI (Antibacterial* OR anti-bacterial* OR “anti bacterial*” OR antimicrobial* OR anti-microbial* OR “anti microbial*” OR “group A streptococcus” OR “streptococcus pyogenes” OR “s. pyogenes” OR “anti streptococc*” OR antistreptococc* OR anti-streptococc* OR “Staphylococcus aureus” OR “s. aureus” OR “staph aureus” OR “anti staphylococc*” OR antistaphylococc* OR anti-staphylococc* OR MRSA OR anti-MRSA OR VRSA OR anti-VRSA) OR AB (Antibacterial* OR anti-bacterial* OR “anti bacterial*” OR antimicrobial* OR anti-microbial* OR “anti microbial*” OR “group A streptococcus” OR “streptococcus pyogenes” OR “s. pyogenes” OR “anti streptococc*” OR antistreptococc* OR anti-streptococc* OR “Staphylococcus aureus” OR “s. aureus” OR “staph aureus” OR “anti staphylococc*” OR antistaphylococc* OR anti-staphylococc* OR MRSA OR anti-MRSA OR VRSA OR anti-VRSA)	367,593
#6	(MM "Streptococcus pyogenes")	9,649

Supplementary Materials S1. Search strategy

#7	(MM "Staphylococcus aureus+") OR (MM "Vancomycin-Resistant Staphylococcus aureus") OR (MM "Methicillin-Resistant Staphylococcus aureus")	50,624
#8	#5 OR #6 OR #7	376,239
#9	#4 AND #8	300

Tl: title, AB: abstract, MM: exact major subject heading, +: explode

Scopus

24.08.2022

No	Search	No of articles
#1	(TITLE-ABS-KEY (*cannabis* OR *hemp* OR *marijuana* OR *phytocannabinoid** OR *cannabinoid** OR *cannabichromene** OR *cannabicyclol** OR *cannabidiol** OR *cbd* OR *cannabielsoin** OR *cannabigerol** OR *cannabinodiol** OR *cannabinol** OR *cannabitriol** OR *tetrahydrocannabi** OR *thc* OR *dronabinol*) AND TITLE-ABS-KEY (*antibacterial** OR *anti-bacterial** OR *"anti bacterial*"* OR *antimicrobial** OR *anti-microbial** OR *"anti microbial*"* OR *"group A streptococcus"* OR *"streptococcus pyogenes"* OR *"s. pyogenes"* OR *"anti streptococc*"* OR *antistreptococc** OR *anti-streptococc** OR *"Staphylococcus aureus"* OR *"s. aureus"* OR *"staph aureus"* OR *"anti staphylococc*"* OR *antistaphylococc** OR *anti-staphylococc** OR *mrsa* OR *anti-mrsa* OR *vrsa* OR *anti-vrsa*))	917

Latin America and Caribbean health Sciences Literature

http://bases.bireme.br/cgi-bin/wxislind.exe/iah/online/?IsisScript=iah/iah.xis&base=LILACS&lang=p&form=A
24.08.2022
Each number responsible for different search

No	Search	No of articles
#1	cannabis OR hemp OR marijuana OR phytocannabinoid$ OR cannabinoid$ OR cannabichromene$ OR cannabicyclol$ OR cannabidiol$ OR cbd OR cannabielsoin$ OR cannabigerol$ OR cannabinodiol$ OR cannabinol$ OR cannabitriol$ OR tetrahydrocannabi$ OR thc OR dronabinol [Words] and antibacterial$ OR anti-bacterial$ OR antimicrobial$ OR anti-microbial$ OR antistreptococc$ OR anti-streptococc$ OR antistaphylococc$ OR anti-staphylococc$ OR MRSA anti-MRSA OR VRSA OR anti-VRSA [Words]	6 Hand searching 1
#2	cannabis OR hemp OR marijuana OR phytocannabinoid$ OR cannabinoid$ OR cannabichromene$ OR cannabicyclol$ OR cannabidiol$ OR cbd OR cannabielsoin$ OR cannabigerol$ OR cannabinodiol$ OR cannabinol$ OR cannabitriol$ OR tetrahydrocannabi$ OR thc OR dronabinol [Words] and anti bacterial$ [Words]	3 Hand searching 0
#3	cannabis OR hemp OR marijuana OR phytocannabinoid$ OR cannabinoid$ OR cannabichromene$ OR cannabicyclol$ OR cannabidiol$ OR cbd OR cannabielsoin$ OR cannabigerol$ OR cannabinodiol$ OR cannabinol$ OR cannabitriol$ OR tetrahydrocannabi$ OR thc OR dronabinol [Words] and anti microbial$ [Words]	1 Hand searching 0
#4	cannabis OR hemp OR marijuana OR phytocannabinoid$ OR cannabinoid$ OR cannabichromene$ OR cannabicyclol$ OR cannabidiol$ OR cbd OR cannabielsoin$ OR cannabigerol$ OR cannabinodiol$ OR cannabinol$ OR cannabitriol$ OR tetrahydrocannabi$ OR thc OR dronabinol [Words] and streptococcus [Words]	1 Hand searching 0
#5	cannabis OR hemp OR marijuana OR phytocannabinoid$ OR cannabinoid$ OR cannabichromene$ OR cannabicyclol$ OR cannabidiol$ OR cbd OR cannabielsoin$ OR cannabigerol$ OR cannabinodiol$ OR cannabinol$ OR cannabitriol$ OR tetrahydrocannabi$ OR thc OR dronabinol [Words] and streptococcus pyogenes [Words]	0
#6	cannabis OR hemp OR marijuana OR phytocannabinoid$ OR cannabinoid$ OR cannabichromene$ OR cannabicyclol$ OR cannabidiol$ OR cbd OR cannabielsoin$ OR cannabigerol$ OR cannabinodiol$ OR cannabinol$ OR cannabitriol$ OR tetrahydrocannabi$ OR thc OR dronabinol [Words] and s. pyogenes [Words]	0
#7	cannabis OR hemp OR marijuana OR phytocannabinoid$ OR cannabinoid$ OR cannabichromene$ OR cannabicyclol$ OR cannabidiol$ OR cbd OR cannabielsoin$ OR cannabigerol$ OR cannabinodiol$ OR cannabinol$ OR cannabitriol$ OR tetrahydrocannabi$ OR thc OR dronabinol [Words] and streptococcal [Words]	0
#8	cannabis OR hemp OR marijuana OR phytocannabinoid$ OR cannabinoid$ OR cannabichromene$ OR cannabicyclol$ OR cannabidiol$ OR cbd OR cannabielsoin$ OR cannabigerol$ OR cannabinodiol$ OR cannabinol$ OR cannabitriol$ OR tetrahydrocannabi$ OR thc OR dronabinol [Words] and Staphylococcus [Words]	0
#9	cannabis OR hemp OR marijuana OR phytocannabinoid$ OR cannabinoid$ OR cannabichromene$ OR cannabicyclol$ OR cannabidiol$ OR cbd OR cannabielsoin$ OR cannabigerol$ OR cannabinodiol$ OR cannabinol$ OR cannabitriol$ OR tetrahydrocannabi$ OR thc OR dronabinol [Words] and s. aureus [Words]	0
#10	cannabis OR hemp OR marijuana OR phytocannabinoid$ OR cannabinoid$ OR cannabichromene$ OR cannabicyclol$ OR cannabidiol$ OR cbd OR cannabielsoin$ OR cannabigerol$ OR cannabinodiol$ OR cannabinol$ OR cannabitriol$ OR tetrahydrocannabi$ OR thc OR dronabinol [Words] and staph aureus [Words]	0
#11	cannabis OR hemp OR marijuana OR phytocannabinoid$ OR cannabinoid$ OR cannabichromene$ OR cannabicyclol$ OR cannabidiol$ OR cbd OR cannabielsoin$ OR cannabigerol$ OR cannabinodiol$ OR cannabinol$ OR cannabitriol$ OR tetrahydrocannabi$ OR thc OR dronabinol [Words] and staphylococcal [Words]	0

Web of Science

24.08.2022
Each number responsible for different search

No	Search	No of articles
#1	cannabis OR hemp OR marijuana OR phytocannabinoid* OR cannabinoid* OR cannabichromene* OR cannabicyclol* OR cannabidiol* OR cbd OR cannabielsoin* OR cannabigerol* OR cannabinodiol* OR cannabinol* OR cannabitriol* OR tetrahydrocannabi* OR thc OR dronabinol **(Topic) and** Antibacterial* OR anti-bacterial* OR antimicrobial* OR anti-microbial* OR antistreptococc* OR anti-streptococc* OR antistaphylococc* OR anti-staphylococc* OR MRSA anti-MRSA	887 (export1)

Supplementary Materials S1. Search strategy

	OR VRSA OR anti-VRSA **(Topic)**	
#2	(TS=(cannabis OR hemp OR marijuana OR phytocannabinoid* OR cannabinoid* OR cannabichromene* OR cannabicyclol* OR cannabidiol* OR cbd OR cannabielsoin* OR cannabigerol* OR cannabinodiol* OR cannabinol* OR cannabitriol* OR tetrahydrocannabi* OR thc OR dronabinol)) AND TS=(anti bacterial*)	579 (export2)
#3	(TS=(cannabis OR hemp OR marijuana OR phytocannabinoid* OR cannabinoid* OR cannabichromene* OR cannabicyclol* OR cannabidiol* OR cbd OR cannabielsoin* OR cannabigerol* OR cannabinodiol* OR cannabinol* OR cannabitriol* OR tetrahydrocannabi* OR thc OR dronabinol)) AND TS=(anti microbial*)	214 (export3)
#4	(TS=(cannabis OR hemp OR marijuana OR phytocannabinoid* OR cannabinoid* OR cannabichromene* OR cannabicyclol* OR cannabidiol* OR cbd OR cannabielsoin* OR cannabigerol* OR cannabinodiol* OR cannabinol* OR cannabitriol* OR tetrahydrocannabi* OR thc OR dronabinol)) AND TS=(group A streptococcus)	14 (export4)
#5	(TS=(cannabis OR hemp OR marijuana OR phytocannabinoid* OR cannabinoid* OR cannabichromene* OR cannabicyclol* OR cannabidiol* OR cbd OR cannabielsoin* OR cannabigerol* OR cannabinodiol* OR cannabinol* OR cannabitriol* OR tetrahydrocannabi* OR thc OR dronabinol)) AND TS=(streptococcus pyogenes)	10 (export5)
#6	(TS=(cannabis OR hemp OR marijuana OR phytocannabinoid* OR cannabinoid* OR cannabichromene* OR cannabicyclol* OR cannabidiol* OR cbd OR cannabielsoin* OR cannabigerol* OR cannabinodiol* OR cannabinol* OR cannabitriol* OR tetrahydrocannabi* OR thc OR dronabinol)) AND TS=(s. pyogenes)	1 (export6)
#7	(TS=(cannabis OR hemp OR marijuana OR phytocannabinoid* OR cannabinoid* OR cannabichromene* OR cannabicyclol* OR cannabidiol* OR cbd OR cannabielsoin* OR cannabigerol* OR cannabinodiol* OR cannabinol* OR cannabitriol* OR tetrahydrocannabi* OR thc OR dronabinol)) AND TS=(anti streptococc*)	35 (export7)
#8	(TS=(cannabis OR hemp OR marijuana OR phytocannabinoid* OR cannabinoid* OR cannabichromene* OR cannabicyclol* OR cannabidiol* OR cbd OR cannabielsoin* OR cannabigerol* OR cannabinodiol* OR cannabinol* OR cannabitriol* OR tetrahydrocannabi* OR thc OR dronabinol)) AND TS=(Staphylococcus aureus)	226 (export8)
#9	(TS=(cannabis OR hemp OR marijuana OR phytocannabinoid* OR cannabinoid* OR cannabichromene* OR cannabicyclol* OR cannabidiol* OR cbd OR cannabielsoin* OR cannabigerol* OR cannabinodiol* OR cannabinol* OR cannabitriol* OR tetrahydrocannabi* OR thc OR dronabinol)) AND TS=(s. aureus)	91 (export9)
#10	(TS=(cannabis OR hemp OR marijuana OR phytocannabinoid* OR cannabinoid* OR cannabichromene* OR cannabicyclol* OR cannabidiol* OR cbd OR cannabielsoin* OR cannabigerol* OR cannabinodiol* OR cannabinol* OR cannabitriol* OR tetrahydrocannabi* OR thc OR dronabinol)) AND TS=(staph aureus)	0
#11	(TS=(cannabis OR hemp OR marijuana OR phytocannabinoid* OR cannabinoid* OR cannabichromene* OR cannabicyclol* OR cannabidiol* OR cbd OR cannabielsoin* OR cannabigerol* OR cannabinodiol* OR cannabinol* OR cannabitriol* OR tetrahydrocannabi* OR thc OR dronabinol)) AND TS=(anti staphylococc*)	120 (export10)

Bibliography search

Supplementary Materials S1. Search strategies

Reference	Selected articles	Exclusion reason
Abichabki et al 2022[1]	Andrade, L. N. et al. Antimicrobial activity of cannabidiol against Eskape pathogens: Inhibitory and bactericidal activity against vancomycin-resistant Enterococcus faecium, vancomycin-intermediate *Staphylococcus aureus*, and methicillin-resistant S. aureus. in ASM MICROBE 2018 (2018).	Article not available
	Abichabki, N. et al. Cannabidiol synergic antimicrobial activity combined with polymyxin B (PB) against PB susceptible and resistant Gram-Negative bacilli. in 30th European Congress of Clinical Microbiology and Infectious Diseases (ECCMID) 2020 (2020)	Abstract not available
	abki, N. et al. In vitro Antibacterial Efcacy of Cannabidiol Plus Polymyxin B: Mixed and Together Against Multidrugresistant and Polymyxin B-resistant Gram-negative Bacilli. in World Microbe Forum Abstracts (2021).	Fulltext not available
	Makabenta, J. M. V. et al. Nanomaterial-based therapeutics for antibiotic-resistant bacterial infections. Nat. Rev. Microbiol. 19, 23–36 (2021)	Review
Blaskovich et al 2021[2]	None	
Farha 2020[3]	Blaskovich, M. A. T.; Kavanagh, A.; Ramu, S.; Levy, S.; Callahan, M.; Thurn, M., Cannabidiol is a Remarkably Active Gram-Positive Antibiotic. In ASM Microbe Conference, San Francisco, California, 2019	Presentation, not available
Kaur et al 2015[4]	**Ali, E.M.M., A.Z.I. Almagboul, S.M.E. Khogali and U.M.A. Gergeir, 2012. Antimicrobial Activity of *Cannabis sativa* L. Chin. Med., 3: 61-64**	Include
	Bhuvaneswari, S., R. Aravind, V. Kaviyarasan, K. Kalaivanan and S.B. Hariram, 2011. A comparative study on antibacterial activity of common weeds. Int. J. Pharm. Biosci., 2: 677-683	No cannabis
	Bonjar, G.H.S., S. Aghighi and A.K. Nik, 2004. Antibacterial and antifungal survey in plants used in indigenous herbal-medicine of South East regions of Iran. J. Biol. Sci., 4: 405-412.	ZOI
	De Boer, H.J., A. Kool, A. Broberg, W.R. Mziray, I. Hedberg and J.J. Levenfors, 2005. Anti-fungal and anti-bacterial activity of some herbal remedies from Tanzania. J. Ethnopharmacol., 96: 461-469.	No cannabis
	Sanguri, S., S. Kapil, P. Gopinathan, F.K. Pandey and T. Bhatnagar, 2012. Comparative screening of antibacterial and antifungal activities of some weeds and medicinal plants leaf extracts: An in-vitro study. Elixir Applied Bot., 47: 8903-8905 https://www.semanticscholar.org/paper/Comparative-screening-of-antibacterial-and-of-some-Sanguri-Kapil/ceac652e68599a44c21d474fd933b3243f4e8e1f	No cannabis
Wassmann et al 2020[5]	None	
Appendino et al 2008[6]	Ferenczy, L.; Gracza, L.; Jakobey, I. An antibacterial preparatum from hemp, Naturwissenschaften 1958, 45, 188	Wrong outcomes
	Krejci, Z. (1958). Hemp (*Cannabis sativa*) antibiotic drugs. II. Method & results of bacteriological experiments & preliminary clinical experience. Die Pharmazie. 13 (3), 155–166	No abstract available
	Rabinovich, A. S.; Aizenman, B. L.; Zelepukha, S. I. [Isolation and investigation of antibacterial properties of preparations from wild hemp (Cannabis ruderalis) growing in the Ukraine], Mikrobiol. Zh 1959, 21, 40–48.	No abstract available
	Schultz, O. E.; Haffner, G. A. Z. [A sedative and antibacterial active agent from the German hemp (*Cannabis sativa*)] Naturforsch. 1959, 14b, 98–100	No abstract available

Supplementary Materials S1. Search strategies

	Gibbons, S. Anti-staphylococcal plant natural products, Nat. Prod. Rep. 2004, 21, 263–277	Review
Chauhan 2017[7]	Zaika LL. Spices and herbs: Their antimicrobial activity and its determination. J Food Saf 1998;9:97-118	No cannabis
	Zavala MA, Perez S, Perez RM. Antimicrobial screening of some medicinal plants. Phytother Res 1997;11:368-71	No cannabis
	Dorman HJ, Deans SG. Antimicrobial agents from plants: Antibacterial activity of plant volatile oils. J Appl Microbiol 2000;88(2):308-16 https://pubmed.ncbi.nlm.nih.gov/10736000/	No cannabis
	Abu-Shanab B, Adwan G, Jarrar N, Abu-Safiya D, Adwan K. Antibacterial activities of some plant extracts utilized in popular medicine in Palestine. Turk J Biol 2004;28:99-102	No cannabis
	Deans SG, Ritchie G. Antibacterial properties of plant essential oils. Int J Food Microbiol 1987;5(2):165-80 https://www.sciencedirect.com/science/article/abs/pii/0168160587900341	No cannabis
	Naveed M, Tahir AK, Izhar A, Adil H, Hamid A, Zaheer UD, et al. In vitro antibacterial activity of *Cannabis sativa* leaf extracts to some selective pathogenicbacterial strains. Int J Biosci 2014;4(4):65-70	ZOI reported
	Raj T, Kumar P, Rathee R, Dubey K. Screening of some medicinal plants for their antimicrobial activities. Int J Pharm Pharm sci 2016;8(5):202-6. Available from: https://www.innovareacademics.in/ journals/index.php/ijpps/article/view/9606/4746	No cannabis
Eisohly 1982[8]	N. S. Hatoum, W. M. Davis, I. W. Waters, M. A. ElSohly, and C E. Turner, Synergism of cannabichromene and CNS depressants in mice, Gen. Pharmacol., 12,351 (1981).	Drug interactions
Muscara 2021[9]	None	
Muscara 2021[10]	Chakraborty, S., Afaq, N., Singh, N., & Majumdar, S. (2018). Antimicrobial activity of *Cannabis sativa*, Thuja orientalis and Psidium guajava leaf extracts against methicillin-resistant *Staphylococcus aureus*. Journal of Integrative Medicine, 16(5), 350–357.	ZOI reported
	Karas, J. A., Wong, L. J. M., Paulin, O. K. A., Mazeh, A. C., Hussein, M. H., Li, J., & Velkov, T. (2020). The antimicrobial activity of cannabinoids. Antibiotics (Basel), 9(7), 406–416	Review
	Nalli, Y., Arora, P., Riyaz-Ul-Hassan, S., Ali, A., & Asif, A. (2018). Chemical investigation of *Cannabis sativa* leading to the discovery of a prenylspirodinone with anti-microbial potential. Tetrahedron Letters, 59 (25), 2470–2472.	IC50 reported
Turner 1981[11]	None	
Van Klingeren 1976[12]	None	
Frassinetti 2020[13]	Bazargani, M., & Rohloff, J. (2016). Antibiofilm activity of essential oils and plant extracts against *Staphylococcus aureus* and Escherichia coli biofilms. Food Control, 61, 156-164 https://www.sciencedirect.com/science/article/abs/pii/S0956713515302152	No cannabis
	Beoletto, V., Oliva, M. de L., Marioli, J. M., Carezzano, M. E. & Demo, M. S. (2016). Antimicrobial natural products against bacterial biofilms. In: K. Kon, & M. Rai. (Eds), Antibiotic Resistance. Mechanisms and new antimicrobial approaches (pp. 291-307). Tokyo (Japan): Academic Press. eBook SBN 9780128036426. https://www.researchgate.net/publication/305366267_Antimicrobial_Natural_Products_Against_Bacterial_Biofilms	Book chapter

Supplementary Materials S1. Search strategies

	Nissen, L., Zatta, A., Stefanini, I., Grandi, S., Sgorbati, B., Biavati, B., & Monti A. (2010). Characterization and antimicrobial activity of essential oils of industrial hemp varieties (*Cannabis sativa* L.). Fitoterapia, 81, 413-419.	No SA or SP
	Nostro, A., Guerrini, A., Marino, A., Tacchini, M., Di Giulio, M., Grandini, A., & Saraçoğlu, H. T. (2016). In vitro activity of plant extracts against biofilm-producing food-related bacteria. International Journal of Food Microbiology, 238, 33–39. https://pubmed.ncbi.nlm.nih.gov/27591384/	No cannabis
Galletta 2020[14]	Klahn, P. Cannabinoids-Promising Antimicrobial Drugs orIntoxicants with Benefits? Antibiotics 2020, 9, 297.	Review
	Silver, L.L.; Bostian, K. Screening of natural products for antimicrobial agents. Eur. J. Clin. Microbiol. Infect. Dis. 1990, 9, 455–461.	review
Martinenghi 2020[15]	Subramani, R.; Narayanasamy, M.; Feussner, K.D. Plant-derived antimicrobials to fight against multi-drug-resistant human pathogens. 3 Biotech 2017, 7, 172. https://pubmed.ncbi.nlm.nih.gov/28660459/	review
	Anjum, M.; Azam, S.; Rehman, P.; Khadim, J. Evaluation of Antimicrobial Activity and Ethnobotanical Study of *Cannabis sativa*. Pure Appl. Biol. 2018, 7, 706–713.	ZOI
Nafis 2019[16]	Chouhan, S., Sharma, K., Guleria, S., 2017. Antimicrobial activity of some essential oils Present status and future perspectives. Medicines 4 (58), 1–21. https://doi.org/10. 3390/medicines4030058.	Review
	Novak, J., Zitterl-Eglseer, K., Deans, S.G., Franz, C.M., 2001. Essential oils of different cultivars of *Cannabis sativa* L. And their antimicrobial activity. Flavour Fragr. J. 16, 259–262. https://doi.org/10.1002/ffj.993.	ZOI
Nigro 2022[17]	None	
Gibbons 2004[18]	None	
Subramani et al 2017[19]	None	
Beoletto et al 2016[20]	None	
Silver and Bostian 1990[21]	None	
Schofs et al 2021[22]	Nascimento GGF, Freitas PC, Silva GL. Antibacterial activity of plant extracts and phytochemicals on antibiotic-resistant bacteria. Braz J Microbiol. 2000;31(4):247–256. https://doi.org/10.1590/ s1517-83822000000400003	no cannabis
	Atef NM, Shanab SM, Negm SI, Abbas YA. Evaluation of the antimicrobial activity of some plant extracts against antibiotic susceptible and resistant bacterial strains causing wound infection. Bull Natl Res Cent. 2019;43(1). https://doi.org/10.1186/s4226 9-019-0184-9	no cannabis
	Chandra H, Bishnoi P, Yadav A, Patni B, Mishra AP, Nautiyal AR. Antimicrobial resistance and the alternative resources with special emphasis on plant-based antimicrobials- a review. Plants. 2017;6(2):16. https://doi.org/10.3390/plants6020016	Review, no reference to cannabis
	Khan BA, Warner P, Wang H. Antibacterial properties of hemp and other natural fibre plants: a review. Bio Res. 2014;9(2):3642–3659. https://doi.org/10.15376/biores.9.2.3642-3659	review

	Wasim K, Haq I, Ashraf M. Antimicrobial studies of the leaf of *Cannabis sativa* L. Pak J Pharm Sci. 1995;8(1):29-38.	ZOI
	Borchardt JR, Wyse DL, Sheaffer CC, et al. Antimicrobial activity of native and naturalized plants of Minnesota and Wisconsin. J Med Plant Res. 2008;2(5):98-110.	ZOI
	Lone TA, Lone RA. Extraction of cannabinoids from *Cannabis sativa* L plant and its potential antimicrobial activity. Univ J Med Dent. 2012;1(4):51-55.	ZOI
	Radwan MM, Elsohly MA, Slade D, Ahmed SA, Khan IA, Ross SA. Biologically active cannabinoids from high-potency *Cannabis sativa*. J Nat Prod. 2009;72(5):906-911. https://doi.org/10.1021/ np900067k	IC50
	Stahl V, Vasudevan K. Comparison of efficacy of cannabinoids versus commercial oral care products in reducing bacterial content from dental plaque: a preliminary observation. Cureus. 2020;12(1):e6809. https://doi.org/10.7759/cureus.6809	Wrong outcomes
	Kosgodage US, Matewele P, Awamaria B, et al. Cannabidiol is a novel modulator of bacterial membrane vesicles. Front Cell Infect Microbiol. 2019;9:324. https://doi.org/10.3389/fcimb.2019.00324	Wrong study design
	Sadgrove NJ, Jones GL. From petri dish to patient: bioavailability estimation and mechanism of action for antimicrobial and immunomodulatory natural products. Front Microbiol. 2019;10:2470. https://doi.org/10.3389/fmicb.2019.02470	Review, no references in bibliography
Klahn 2020[23]	Ferenczy, L. Antibacterial Substances in Seeds. Nature 1956, 178, 639–640.	ZOI
	Monika, K.N.; Kaur, M. Antimicrobial analysis of leaves of *Cannabis sativa*. J. Sci. 2014, 4, 123–127	ZOI
	**Sarmadyan, H.; Solhi, H.; Hajimir, T.; Najarian-Araghi, N.; Ghaznavi-Rad, E. Determination of the Antimicrobial Effects of Hydro-Alcoholic Extract of *Cannabis Sativa* on Multiple Drug Resistant Bacteria Isolated from Nosocomial Infections. Iran. J. Toxicol. 2014, 7, 967–972**	include
	Fathordoobady, F.; Singh, A.; Kitts, D.D.; Singh, A.P. Hemp (*Cannabis sativa* L.) Extract: Anti-Microbial Properties, Methods of Extraction, and Potential Oral Delivery. Food Rev. Int. 2019, 664–684.	review
	Verma, R.S.; Padalia, R.C.; Verma, S.K.; Chauhan, A.; Darokar, M.P. The essential oil of' bhang' (*Cannabis sativa* L.) for non-narcotic applications. Curr. Sci. 2014, 107, 645–650.	ZOI
	Raina, S.; Thakur, A.; Sharma, A.; Pooja, D.; Minhas, A.P. Bactericidal activity of *Cannabis sativa* phytochemicals from leaf extract and their derived Carbon Dots and Ag@Carbon Dots. Mater. Lett. 2019, 262, 127122.	Wrong outcomes
	Das, B.; Mishra, P.C. Antibacterial analysis of crude extracts from the leaves of Tagetes erecta and *Cannabis sativa*. Int. J. Environ. Sci. 2012, 2, 1605–1609.	ZOI, no SA, SP
	Mkpenie, V.N.; Essien, E.E.; Udoh, I.I. Effect of extraction conditions on total polyphenol contents, antioxidant and antimicrobial activities of *Cannabis sativa* L. Electron. J. Environ. Agric. Food Chem. 2012, 11, 300–307	ZOI
	Kakar, S.A.; Tareen, R.B.; Azam Kakar, M.; Jabeen, H.; Kakar, S.U.R.; Al-Kahraman, Y.M.S.A.; Shafee, M. Screening of antibacterial activity of four medicinal plants of Balochistan-Pakistan. Pakistan J. Bot. 2012, 44, 245–250	no cannabis
	Mathur, P.; Singh, A.; Srivastava, V.R.; Singh, D.; Mishra, Y. Antimicrobial activity of indigenous wildly growing plants: Potential source of green antibiotics. African J. Microbiol. Res. 2013, 7, 3807–3815	ZOI
	Tandon, C.; Mathur, P. Antimicrobial Efficacy of *Cannabis sativa* L. (Bhang): A Comprehensive Review. Int. J. Pharm. Sci. Rev. Res. 2017, 44, 94–100	review
	Glodowska, M.; Łyszcz, M. *Cannabis sativa* L. and its antimicrobial properties-A review. In Badania i Rozwój	review

Supplementary Materials S1. Search strategies

	Młodych Naukowców w Polsce–Agronomia i Ochrona Ro´slin; Le´sny, J., Chojnicki, B., Panfil, M., Ny´ckowiak, J., Eds.; Młodzi Naukowcy: Pozna ´n, Poland, 2017; pp. 77–82	
Chouhan *et al.* 2021[24]	None	
Fathordoobady *et al.* 2019[25]	Yasmeen, R.; Hashmi, A. S.; Anjum, A. A.; Saeed, S.; Muhammad, K. Antibacterial Activity of Indigenous Herbal Extracts against Urease Producing Bacteria. J. Anim. Plant Sci. 2012, 22 (2), 416–419.	no SP or SA
Glodowska and Lyszcz 2016[26]	Gallucci MN, Oliva M, Casero C, et al. (2009) Antimicrobial combined action of terpenes against the food-borne microorganisms Escherichia coli, *Staphylococcus aureus* and Bacillus cereus. Flavour and Fragrance J, Vol. 24(6): 348–54.	No cannabis
Karas *et al.* 2020	**Vu, T.T.; Kim, H.; Tran, V.K.; Le Dang, Q.; Nguyen, H.T.; Kim, H.; Kim, I.S.; Choi, G.J.; Kim, J.C. In vitro antibacterial activity of selected medicinal plants traditionally used in Vietnam against human pathogenic bacteria. BMC Complement. Altern. Med. 2016, 16, 32**	Include
	Lelario, F.; Scrano, L.; Franchi, S.D.; Bonomo, M.G.; Salzano, G.; Milan, S.; Milella, L.; Bufo, S.A. Identification and antimicrobial activity of most representative secondary metabolites from different plant species. Chem. Biol. Technol. Agric. 2018, 5, 1–12.	no SP or SA
	Mikulcová, V.; Kašpárková, V.; Humpolí˘cek, P.; Bu ˇnková, L. Formulation, Characterization and properties of hemp seed oil and its emulsions. Molecules 2017, 22, 700	ZOI
	Nadir, I.; Rana, N.F.; Ahmad, N.M.; Tanweer, T.; Batool, A.; Taimoor, Z.; Riaz, S.; Ali, S.M. Cannabinoids and terpenes as an antibacterial and antibiofouling promotor for pes water filtration membranes. Molecules 2020, 25, 691	ZOI
	Feldman, M.; Smoum, R.; Mechoulam, R.; Steinberg, D. Antimicrobial potential of endocannabinoid and endocannabinoid-like compounds against methicillin-resistant *Staphylococcus aureus*. Sci. Rep. 2018, 8, 17696	Wrong design
	Feldman, M.; Smoum, R.; Mechoulam, R.; Steinberg, D. Potential combinations of endocannabinoid/ endocannabinoid-like compounds and antibiotics against methicillin-resistant *Staphylococcus aureus*. PLoS ONE 2020, 15, e0231583.	Wrong design
Tandon and Mathur 2017[27]	Nasrullah, Suliman, Rahman K, Ikram M, Nisar M, Khan I, Screening of antibacterial activity of medicinal plants, Int J Pharm Sci Rev Res, 14, 2012, 25-29.	ZOI
Iseppi *et al.* 2019[28]	Pellati, F.; Brighenti, V.; Sperlea, J.; Marchetti, L.; Bertelli, D.; Benvenuti, S. New methods for the comprehensive analysis of bioactive compounds in *Cannabis sativa* L. (hemp). Molecules 2018, 23, 2639	no SP or SA
	Russo, E.B. Taming THC: Potential cannabis synergy and phytocannabinoid-terpenoid entourage effects. Br. J. Pharmacol. 2011, 163, 1344–1364.	Review
Ali *et al.* 2012[29]	None	
Sarmadyan *et al.* 2014[30]	Radosevi, Cacute A, KUPINI, Cacute M,GRLI, Cacute L. Antibiotic activity of various types of Cannabis resin. 1962;8(195):1007-9 https://www.nature.com/articles/1951007a0	wrong design

Supplementary Materials S1. Search strategies

	Sumthong P. Antimicrobial compounds as side products from the agricultural processing industry: Division of Pharmacognosy, Section of Metabolomics, Institute of Biology, Faculty of Science, Leiden University; 2007.p. 15-32	wrong design
Vu *et al.* 2016[31]	Tekwu EM, Pieme AC, Beng VP. Investigations of antimicrobial activity of some Cameroonian medicinal plant extracts against bacteria and yeast with gastrointestinal relevance. J Ethnopharmacol. 2012;142:265–73 https://www.sciencedirect.com/science/article/abs/pii/S037887411200298X?via%3Dihub	No cannabis
	Khan UA, Rahman H, Niaz Z, Qasim M, Khan J, Tayyaba, *et al.*. Antibacterial activity of some medicinal plants against selected human pathogenic bacteria. Eur J Microbiol Immunol. 2013;3:272–4.	No cannabis
	Fankam AG, Kuiate JR, Kuete V. Antibacterial activities of Beilschmiedia obscura and six other Cameroonian medicinal plants against multi-drug resistant Gram-negative phenotypes. BMC Complement Altern Med. 2014;14:241	No cannabis
	Cowan MM. Plant products as antimicrobial agents. Clin Microbiol Rev. 1999;2:564–82	Review, no cannabis in bibliography
	Bussmann RW, Malca-Garcia G, Glenn A, Sharon D, Chait G, Diaz D, *et al.*. Minimum inhibitory concentrations of medicinal plants used in Northern Peru as antibacterial remedies. J Ethnopharmacol. 2010;32:1–8	No cannabis
	Moura-Costa GF, Nocchi SR, Ceole LF, de Mello JCP, Nakamura CV, Filho BPD, *et al.*. Antimicrobial activity of plants used as medicinals on an indigenous reserve in Rio das Cobras, Parana. Brazil J Ethnopharmacol. 2012;143:631–8	No cannabis
	Fomogne-Fodjo MCY, Vuuren SV, Ndinteh DT, Krause RWM, Olivier DK. Antibacterial activity of plant from Central Africa used traditionally by the Bakola pygmies for treating respiratory and tuberculosis-related symptoms. J Ethnopharmacol. 2014;115:123–31.	No cannabis
	Ocheng F, Bwanga F, Joloba M, Borg-Karlson AK, Gustafsson A, Obua C. Antibacterial activity of extracts from Ugandan medicinal plants used for oral care. J Ethnopharmacol. 2014;155:852–5	No cannabis
	Robles-Zepeda RE, Coronado-Aceves EW, Velazquez-Contreras CA, Ruiz Bustos E, Navarro-Navarro M, Garibay-Escobar A. In vitro anti-mycobacterial activity of nine medicinal plants used by ethnic groups in Sonora, Mexico. BMC Complement Altern Med. 2013;13:329	No cannabis
	Khan N, Abbasi AM, Dastagir G, Nazir A, Shah GM, Shah MM, *et al.*. Ethnobotanical and antimicrobial study of some selected medicinal plants used in Khyber Pakhtunkhwa (KPK) as potential source to cure infectious diseases. BMC Complement Altern Med. 2014;14:122	No cannabis
Zengin *et al.* 2018[32]	Khan, S.; Ur-Rehman, T.; Mirza, B.; Ul-Haq, I.; Zia, M. Antioxidant, antimicrobial, cytotoxic and protein kinase inhibition activities of fifteen traditional medicinal plants from Pakistan. Pharm. Chem. J. 2017, 51, 391–398	Wrong outcomes

ZOI: zone of inhibition, SA: *Staphylococcus aureus*, SP: *Streptococcus pyogenes*

Supplementary Materials S2. Details of rejected articles

Following are the details of articles rejected at the full-text screening.

Thirteen studies were excluded due to wrong study design[33-45]. Twenty-nine studies were excluded because wrong outcomes[46-74]. Three articles were excluded because they did not have full texts[75-77].

Supplementary Table S1. Descriptive characteristics of the included studies (n=24)

Supplementary Table S1. Descriptive characteristics of the included studies (n=24).

Reference, study origin	Study objective	Names and details of cannabinoids examined	Sources of cannabinoids examined	Description of quantitative antibacterial studies conducted	Study limitations	Study quality
**Abichabki *et al.* 2022[1] Brazil, Spain**	To evaluate the in vitro antibacterial activity of ultrapure CBD against a wide diversity of bacteria	CBD	Purchase:<br>(BSPG-Pharm, Sandwich, UK, purity=99.6%)	**MIC**<br>*Assay:* broth microdilution (EUCAST and ISO standards)<br>*Final inoculum:* not given<br>*Culture medium:* (for all *S. aureus*: CAMHB, *S. aureus* ATCC 29213 also tested in MH-F, *S. pyogenes* in MH-F)<br>*Incubation:* not given<br>*Plate type:* 96-well microplates polystyrene, round bottom, non-treated<br>*Solvent (stock solution):* MeOH	-	1
**Ali *et al.* 2012[29] Sudan**	To evaluate the antimicrobial activity of the extracts of *C. sativa* grown in Sudan against standard bacteria and the fungi	Seeds and the whole plant of *C. sativa* were sequentially extracted into petroleum ether and MeOH. MeOH extract was used to test MIC	Extracted	**MIC**<br>*Assay:* Agar plate dilution method<br>*Final inoculum:* not given<br>*Culture medium:* not given<br>*Incubation:* not given<br>*Solvent (stock solution):* not given	MIC method was only given in the abstract, no description in methodology.	3
**Appendino *et al.* 2008[6] Italy, UK**	To investigate the antibacterial profile of five major cannabinoids; of their alkylation and acylation products; and of a selection of their carboxylic precursors (pre-cannabinoids) and synthetic positional isomers (abnormal cannabinoids)	CBC, CBDA[78], CBD, CBGA[79], CBG, CBGA methyl ester, CBN, THCAA[3], Δ9-THC, abn-CBD, abn-CBG, carmagerol, CBD-di-Ac[80], CBDM[81], CBD dimethyl ether[81], CBDA methyl ester[82], phenethyl ester of CBDA, CBG-di-Ac, CBG monomethyl ether, CBG dimethyl ether, phenethyl ester of CBGA, 3'-bis-prenyl CBD	Isolated from *C. sativa*: THC, CBD, CBDA, CBG, CBGA, THCAA<br><br>Semi-synthesis: CBN, CBD-di-Ac, CBD monomethyl ether, CBD dimethyl ether, CBDA methyl ester, phenethyl ester of CBDA, CBGA methyl ester, CBG-di-Ac, CBG monomethyl ether, CBG dimethyl ether, phenethyl ester of CBGA<br><br>Synthesis:<br>CBG, CBC, abn-CBD, abn-CBG, 3'-bis-prenyl	**MIC**<br>*Assay:* broth microdilution<br>*Final inoculum:* 5 x 10^5 CFU/mL<br>*Culture medium:* CAMHB<br>*Incubation:* 37 °C for 18 h<br>*Plate type:* Nunc 96-well microtiter<br>*Solvent (stock solution):* DMSO	-	1

Supplementary Table S1. Descriptive characteristics of the included studies (n=24)

			CBD			
**Blaskovich *et al.* 2021[2] Australia, New Zealand, US**	To evaluate the antimicrobial activity of cannabinoids	7-hydroxycannabidiol, 7-nor-7-carboxycannabidiol, 7-nor-7-carboxy-cannabidivarin, 7-nor-7-hydroxymethyl-cannabidivarin, CBD, CBDA, CBDV, CBG, CBGA, CBNA, MTC-002, MTC-005, MTC-007, MTC-008, MTC-009, MTC-011, MTC-012, MTC-013, MTC-014, MTC-017, MTC-018, (-)-Δ9-THC, THCV, THCVA, THCA-A, (-)-Δ8-THC	Purchase:<br>CBD (from AMRI), 7-hydroxycannabidiol, 7-nor-7-carboxycannabidiol, CBDA, CBG, CBGA, CBNA, THCV, THCVA, (-)-Δ-9-THC, THCA-A, (-)-Δ8-THC (from Kinesis Australia Pty Ltd., distributor Cerilliant Corporation)<br><br>Synthesis:<br>CBDV, 7-nor-7-hydroxymethyl-cannabidivarin (purity=98.3%), 7-nor-7-carboxy-cannabidivarin (100%), MTC-002 (100%), MTC-005 (97.2%), MTC-007 (99.0%), MTC-008 (99.6%), MTC-009 (99.8%), MTC-011 (98.5%), MTC-012 (99.0%), MTC-013 (99.6%), MTC-014 (99.8%), MTC-017 (97.6%), MTC-018 (98.9%)	**MIC**<br>**Aerobic assay**<br>*Assay:* broth microdilution (CLSI)<br>*Final inoculum:* 5 x 10^5 CFU/mL<br>*Culture medium:* CAMHB (for *S. aureus*, MRSA and VRSA), CAMHB+ 3% LHB (*S. pyogenes*)<br>*Incubation:* 37 °C for 20 h<br>*Plate type:* flat bottom 96-well polystyrene (Corning, Cat. No. 3370)<br>*Solvent (stock solution):* DMSO<br><br>**Anarobic assay**<br>*Assay:* broth microdilution<br>*Final inoculum:* 5 x 10^5 CFU/mL<br>*Culture medium:* BHI broth with 1% cysteine<br>*Incubation:* 10%CO_2/5% H_2, 37 °C for 48 h<br>*Plate type:* flat bottom 96-well polystyrene (Corning, Cat. No. 3370)<br>*Solvent (stock solution):* DMSO	-	1
**Chauhan *et al.* 2017[7] India**	To evaluate the antibacterial effect of Himalayan medicinal plants on several human pathogens	MeOH extract of *C. sativa* leaves	Extracted	**MIC**<br>*Assay:* broth microdilution<br>*Final inoculum:* not given<br>*Culture medium:* NB<br>*Incubation:* 37°C for 24 h<br>*Plate type:* not given<br>*Solvent (stock solution):* DMSO	-	3
**Eisohly *et al.* 1982[8] US**	To describe the synthesis and evaluate antimicrobial activities of CBC homologs, analogs, and isomers	CBC[83], CBC-C_0[11,84], CBC-C_1[11,84], iso-CBC-C_0[11], 2-Methyl-2-(4'-methylpentyl)-5-hydroxy-7-methylchroman, CBG-C_1, 2-	Synthesis<br>CBC- C_1 (>95% pure)	**MIC**<br>*Assay:* Broth macrodilution<br>*Final inoculum:* not given<br>*Culture medium:* not given<br>*Incubation:* 24 h and 48 h	The study was conducted before CLSI guidelines	3

Supplementary Table S1. Descriptive characteristics of the included studies (n=24)

	and that of CBG-C_1, iso-CBG-C_1, and tetrahydrocannabigerol-C_1	Tetrahydrogeranyl-5-methyl Resorcinol, 2-Methyl-2-(4'-methylpent-3'-enyl)-5-hydroxy-7-pentadec-8''-enylchromene, 2-Methyl-2-(4'-methylpent-3'-enyl)–5,7-dihydroxychromene		*Solvent (stock solution):* not given	establish	
**Farha *et al.* 2020[3] Canada, Egypt**	To investigate the antibacterial, anti-biofilm and anti-persister activity of a variety of cannabinoids	CBC, CBCA, CBD, CBDA, CBDV, CBDVA, CBG, CBGA, CBL, CBN, (-)Δ8-THC, (-)Δ9-THC, exo-THC, THCAA, THCV, THCVA, (±)11-nor-9-carboxy-Δ9-THC, (±)11-hydroxy-Δ9-THC	Synthesis:<br>CBG<br><br>Purchase:<br>All other cannabinoids (Sigma, Oakville, ON, Canada)	**MIC**<br>*Assay:* broth microdilution (CLSI)<br>*Final inoculum:* not given<br>*Culture medium:* CAMHB<br>*Incubation:* not given<br>*Plate type:* not given<br>*Solvent (stock solution):* not given	-	3
**Frassinetti *et al.* 2020[13] Italy**	To evaluate the inhibitory activity of *C. sativa* L. seeds extract, cultivar Futura 75, on the biofilm formation by *S. aureus*	80% EtOH extract of *Cannabis sativa* L. cultivar Futura 75 seeds	Extracted	**MIC**<br>*Assay:* broth microdilution<br>*Final inoculum:* 1-5 x 10^5 CFU/mL<br>*Culture medium:* MHB<br>*Incubation:* 37 °C for 24 h<br>*Plate type:* not given<br>*Solvent (stock solution):* sterile water	-	3
**Galletta *et al.* 2020[14] Australia**	To characterise the ability of the CBCA and its related synthetic analogues to successfully inhibit the growth of MRSA and other clinically relevant pathogenic bacteria	(±)-CBCA, (±)-CBCM, (±)-CBCTFA, (±)-CBLM, CBDVM	Synthesis<br>>95% purity	**MIC**<br>*Assay:* broth microdilution<br>*Final inoculum:* not given<br>*Culture medium:* LB broth<br>*Incubation:* 37 °C for overnight<br>*Plate type:* not given<br>*Solvent (stock solution):* DMSO	-	3
**Iseppi *et al.* 2019[28] Italy**	Aimed at the phytochemical characterization of 17 hemp EO belonging to different varieties, together with the evaluation of their antibacterial activity	EO of *C. sativa* (from different fibre-type varieties, from inflorescences or the whole plants)<br>CBD	Extracted:<br>EO<br><br>Purchase:<br>CBD (Cerilliant, Round Rock, TX, US)	**MIC**<br>*Assay:* Broth microdilution (CLSI)<br>*Final inoculum:* 10^6 CFU/mL<br>*Culture medium:* NB<br>*Incubation:* 37 °C for 24 h<br>*Plate type:* not given<br>*Solvent (stock solution):* MeOH (CBD)	Higher final inoculum concentration	1
**Kaur *et al.* 2015[4] India**	To test the efficacy of some common weeds extracts against the	Separate extractions of EtOH, MeOH, acetone and water of *C. sativa* leaves	Extracted	**MIC**<br>*Assay:* modified agar well diffusion method	MIC: lowest concentration of the extract	3

Supplementary Table S1. Descriptive characteristics of the included studies (n=24)

	bacterial and fungal pathogens			*Final inoculum:* 5 x 10^5 CFU/mL<br>*Culture medium:* NA<br>*Incubation:* 37°C for 24 h<br>*Solvent (stock solution):* DMSO	that completely inhibited the growth of the microbe, showed by a clear zone of inhibition (>12 mm)<br><br>This method is not described in CLSI	
**Martinenghi *et al.* 2020[15] Denmark**	To have an overview of the antimicrobial effect of CBDA, and CBD	CBD, CBDA	Isolated from inflorescences of fiber type *C. sativa* L	**MIC**<br>*Assay:* broth microdilution (CLSI)<br>*Final inoculum:* not given<br>*Culture medium:* not given<br>*Incubation:* not given<br>*Plate type:* not given<br>*Solvent (stock solution):* EtOAc (CBDA) and MeOH (CBD)	-	1
**Muscara *et al.* 2021[9] Italy**	To evaluate and compare the phytochemical profile, antioxidant and antimicrobial properties of two different standardized extracts obtained from dried flowering tops (as such and after hydrodistillation of the essential oil) of a non-psychotropic CBD-rich *C. sativa* L. var. *fibrante*	0.1% acetic acid/hexane extract of dried flowering tops *C. sativa* L. var. *fibrante* as such and after hydrodistillation of the essential oil	Extracted	**MIC**<br>*Assay:* broth microdilution (CLSI)<br>*Final inoculum:* not given<br>*Culture medium:* not given<br>*Incubation:* 20 h<br>*Plate type:* not given<br>*Solvent (stock solution):* DMSO<br><br>**MBC** (CLSI)<br>*Culture medium:* MHA<br>*Incubation:* 24 h	-	1
**Muscara *et al.* 2021[10] Italy**	To chemically and biologically characterize extracts of *C. sativa*	0.1% acetic acid/hexane extract of dried flowering tops of *C. sativa* Chinese accession (G-309) as such and after hydrodistillation of the essential oil	Extracted	**MIC**<br>*Assay:* broth microdilution (CLSI)<br>*Final inoculum:* not given<br>*Culture medium:* not given<br>*Incubation:* 24 h<br>*Plate type:* not given	-	3

Supplementary Table S1. Descriptive characteristics of the included studies (n=24)

				Solvent (stock solution): not given<br><br>**MBC** (CLSI)<br>*Culture medium:* not given<br>*Incubation:* 24 h at 37°C		
**Nafis *et al.* 2019[16] Morocco, US**	To characterize the EO of Moroccan *C. sativa*, to evaluate its antioxidant and antimicrobial properties, and to evaluate the possible synergistic combination of this EO with ciprofloxacin and fluconazole against some resistant bacteria and clinic pathogenic yeasts	EO from the aerial parts of *C. sativa*	Extracted	**MIC**<br>*Assay:* broth microdilution (CLSI)<br>*Final inoculum:* 1 x 10^5 CFU/mL<br>*Culture medium:* not given<br>*Incubation:* not given<br>*Plate type:* not given<br>*Solvent (stock solution):* DMSO	-	3
**Nigro *et al.* 2022[17] Italy**	To optimize fast and low-cost purification strategies of CBDA, and a deep investigation on its nutraceutical and cosmeceutical properties	CBDA	Extracted from *C. sativa*	**MIC**<br>*Assay:* modified version of the broth microdilution (CLSI)<br>*Final inoculum:* 10^5 CFU/mL<br>*Culture medium:* BHI broth<br>*Incubation:* 37 °C for 3-5 h<br>*Plate type:* not given<br>*Solvent (stock solution):* not given<br><br>**MBC** (CLSI)<br>Method not described	inoculum was shaken at 225 rpm for 15-18 h before inoculation<br><br>incubated for considerably lesser time	3
**Pellegrini *et al.* 2021[85] Italy**	To characterize *C. sativa* L. cv Futura 75 inflorescences, cultivated in the Abruzzo territory, for their volatile fraction, to investigate the essential oil extracted from these inflorescences for the antioxidant potentialities, terpenic	EO of *C. sativa* 'Futura 75' inflorescence	Extracted	**MIC**[86]<br>*Assay:* broth microdilution (CLSI)<br>*Final inoculum:* 5 x 10^5 CFU/mL<br>*Culture medium:* TSB<br>*Incubation:* 37°C for 48 h<br>*Solvent (stock solution):* Phosphate Buffer Saline 50 mM pH 7.0 and Tween 80 (1%)	Considerably longer incubation time	3

Supplementary Table S1. Descriptive characteristics of the included studies (n=24)

	profile and antibacterial activity			**MBC**[86]<br>*Culture medium:* TSA<br>*Incubation:* 37°C		
**Sarmadyan *et al.* 2014[30] Iran**	To investigate the antibacterial property of *C. sativa* on standard and resistant bacteria with multiple forms of resistance which are increasingly found in hospitals	Hydro-alcoholic extract of *C. sativa*	Extracted	**MIC**<br>*Assay:* broth macrodilution<br>*Final inoculum:* 1.5 x 10^8 CFU/mL<br>*Culture medium:* not given<br>*Incubation:* 37°C for 24 h<br>*Solvent (stock solution):* DMSO	High inoculum size, negative control mis-interpreted as the positive control	1
**Schuetz *et al.* 2021[87] Canada, US**	To characterize CBG's activity and safety profile for use on skin	CBD, CBG	Yeast fermentation: CBG	MIC<br>no details available	Data extracted from abstract and the poster, no details available on the method	3
**Turner and Elsohly 1981[11] US**	To determine antiinflammatory, antibacterial, and antifungal properties of CBC and its homologs and isomers	CBC, CBC-C_0, CBC-C_1, iso-CBC-C_0	Synthesis	**MIC**<br>*Assay:* broth macrodilution<br>*Final inoculum:* one loop-full of a 1:10 dilution of the 24-hour-old broth culture of the test organisms in sterile water<br>*Culture medium:* Eugon broth (50 mL)<br>*Incubation:* 24 h and 48 h<br>*Solvent (stock solution):* not given	-	3
**van Klingeren and Ten 1976[12] The Netherlands**	To quantitative determinations of the bacteriostatic and bactericidal action of purified delta-9-THC and CBD	CBD, Δ9-trans-THC	Isolate:<br>CBD (method not given)<br><br>Obtain:<br>Δ9-trans-THC (Bureau of Narcotic Drugs of the United Nations)	**MIC**<br>*Assay:* Agar dilution method<br>*Final inoculum:* 10^3 CFU/mL<br>*Culture medium:* NA, horse blood agar (4% horse serum)<br>*Incubation:* 37°C overnight<br>*Solvent (stock solution):* 70% EtOH	Low final inoculum	3

Supplementary Table S1. Descriptive characteristics of the included studies (n=24)

**Vu *et al.* 2016[31] Vietnam, Korea**	To validate the traditional use of selected medicinal plants against common bacteria by evaluating their in vitro antibacterial activity	MeOH extract of leaves and branches of *C. sativa*	Extract	**MIC**<br>*Assay:* broth microdilution<br>*Final inoculum:* 10^5 CFU/mL before adding the extract<br>*Culture medium:* NB<br>*Incubation:* 37 °C for 24–h<br>*Plate type:* sterile 96-well plates<br>*Solvent (stock solution):* DMSO<br><br>**MBC**<br>*Culture medium:* NA<br>*Incubation:* 37 °C, 24–48 h	Low final inoculum, Volume of extract, and dilution of the inoculum were not given. Therefore, exact final inoculum is unknown. Inoculum was added and then the extracts were added.	1
**Wassmann *et al.* 2020[5] Denmark**	To characterise CBD as a potential helper compound against resistant bacteria in combination with the cyclic peptide antibiotic BAC	CBD	Purchase (Sigma Aldrich)	**MIC**<br>*Assay:* broth microdilution<br>*Final inoculum:* 5 x 10^5 CFU/mL<br>*Culture medium:* BHI or MHB<br>*Incubation:* 37 °C for 16–22hours with agitation<br>*Plate type:* 96-well plates (Nunc A/S or Sarstedt)<br>*Solvent (stock solution):* EtOH	Plates were agitated	3
**Zengin *et al.* 2018[32] Italy, Turkey**	To explore chemically and biologically both the essential oil and the aromatic water of Italian *C. sativa* L.	EO of *C. sativa* L. Futura 75 cultivar (aerial parts consisting of leaves, inflorescences, and thinner residues of stem)	Extracted 10% (v/v) solutions in EtOH used in testing	**MIC**<br>*Assay:* broth microdilution (NCCLS guideline)<br>*Final inoculum:* 1.0 × 10^5 CFU/mL<br>*Culture medium:* MHB<br>*Incubation:* 37 °C for 24 h<br>*Plate type:* 96-well polystyrene microtitre plates (Eppendorf, Hamburg, Germany)<br>*Solvent (stock solution):* 10% (v/v) solutions in EtOH<br>**MBC**<br>*Culture medium:* not given<br>*Incubation:* not given	-	1

Supplementary Table S2. PRISMA 2020 Checklist

Section and Topic	Item #	Checklist item	Location where item is reported
**TITLE**			
Title	1	Identify the report as a systematic review.	title
**ABSTRACT**			
Abstract	2	See the PRISMA 2020 for Abstracts checklist.	abstract
**INTRODUCTION**			
Rationale	3	Describe the rationale for the review in the context of existing knowledge.	Introduction
Objectives	4	Provide an explicit statement of the objective(s) or question(s) the review addresses.	Introduction
**METHODS**			
Eligibility criteria	5	Specify the inclusion and exclusion criteria for the review and how studies were grouped for the syntheses.	Methods
Information sources	6	Specify all databases, registers, websites, organisations, reference lists and other sources searched or consulted to identify studies. Specify the date when each source was last searched or consulted.	Methods, Supplementary material S1
Search strategy	7	Present the full search strategies for all databases, registers and websites, including any filters and limits used.	Supplementary material S1
Selection process	8	Specify the methods used to decide whether a study met the inclusion criteria of the review, including how many reviewers screened each record and each report retrieved, whether they worked independently, and if applicable, details of automation tools used in the process.	Methods
Data collection process	9	Specify the methods used to collect data from reports, including how many reviewers collected data from each report, whether they worked independently, any processes for obtaining or confirming data from study investigators, and if applicable, details of automation tools used in the process.	Methods
Data items	10a	List and define all outcomes for which data were sought. Specify whether all results that were compatible with each outcome domain in each study were sought (e.g. for all measures, time points, analyses), and if not, the methods used to decide which results to collect.	Methods
	10b	List and define all other variables for which data were sought (e.g. participant and intervention characteristics, funding sources). Describe any assumptions made about any missing or unclear information.	Methods
Study risk of bias assessment	11	Specify the methods used to assess risk of bias in the included studies, including details of the tool(s) used, how many reviewers assessed each study and whether they worked independently, and if applicable, details of automation tools used in the process.	Methods
Effect measures	12	Specify for each outcome the effect measure(s) (e.g. risk ratio, mean difference) used in the synthesis or presentation of results.	Methods
Synthesis methods	13a	Describe the processes used to decide which studies were eligible for each synthesis (e.g. tabulating the study intervention characteristics and comparing against the planned groups for each synthesis (item #5)).	Methods, results
	13b	Describe any methods required to prepare the data for presentation or synthesis, such as handling of missing summary statistics, or data conversions.	Methods, results
	13c	Describe any methods used to tabulate or visually display results of individual studies and syntheses.	Methods, results
	13d	Describe any methods used to synthesize results and provide a rationale for the choice(s). If meta-analysis was performed, describe the model(s), method(s) to identify the presence and extent of statistical heterogeneity, and software package(s) used.	Methods, results
	13e	Describe any methods used to explore possible causes of heterogeneity among study results (e.g. subgroup analysis, meta-regression).	Methods, results
	13f	Describe any sensitivity analyses conducted to assess robustness of the synthesized results.	Not applicable
Reporting bias assessment	14	Describe any methods used to assess risk of bias due to missing results in a synthesis (arising from reporting biases).	Not applicable

Supplementary Table S2. PRISMA 2020 Checklist

Section and Topic	Item #	Checklist item	Location where item is reported
Certainty assessment	15	Describe any methods used to assess certainty (or confidence) in the body of evidence for an outcome.	Not applicable
**RESULTS**			
Study selection	16a	Describe the results of the search and selection process, from the number of records identified in the search to the number of studies included in the review, ideally using a flow diagram.	PRISMA chart, results
	16b	Cite studies that might appear to meet the inclusion criteria, but which were excluded, and explain why they were excluded.	Supplementary material S2
Study characteristics	17	Cite each included study and present its characteristics.	results, tables 1-6, Supplementary Table S1
Risk of bias in studies	18	Present assessments of risk of bias for each included study.	Supplementary material S1
Results of individual studies	19	For all outcomes, present, for each study: (a) summary statistics for each group (where appropriate) and (b) an effect estimate and its precision (e.g. confidence/credible interval), ideally using structured tables or plots.	Results, figures 2-4, Supplementary material S1
Results of syntheses	20a	For each synthesis, briefly summarise the characteristics and risk of bias among contributing studies.	Not applicable
	20b	Present results of all statistical syntheses conducted. If meta-analysis was done, present for each the summary estimate and its precision (e.g. confidence/credible interval) and measures of statistical heterogeneity. If comparing groups, describe the direction of the effect.	Results, figures 2-4
	20c	Present results of all investigations of possible causes of heterogeneity among study results.	Results
	20d	Present results of all sensitivity analyses conducted to assess the robustness of the synthesized results.	Results
Reporting biases	21	Present assessments of risk of bias due to missing results (arising from reporting biases) for each synthesis assessed.	Results
Certainty of evidence	22	Present assessments of certainty (or confidence) in the body of evidence for each outcome assessed.	Results
**DISCUSSION**			
Discussion	23a	Provide a general interpretation of the results in the context of other evidence.	Discussion
	23b	Discuss any limitations of the evidence included in the review.	Discussion
	23c	Discuss any limitations of the review processes used.	Discussion
	23d	Discuss implications of the results for practice, policy, and future research.	Discussion
**OTHER INFORMATION**			
Registration and protocol	24a	Provide registration information for the review, including register name and registration number, or state that the review was not registered.	Method
	24b	Indicate where the review protocol can be accessed, or state that a protocol was not prepared.	Method
	24c	Describe and explain any amendments to information provided at registration or in the protocol.	Not applicable
Support	25	Describe sources of financial or non-financial support for the review, and the role of the funders or sponsors in the review.	Acknowledgement
Competing interests	26	Declare any competing interests of review authors.	Competing interests

Supplementary Table S2. PRISMA 2020 Checklist

Section and Topic	Item #	Checklist item	Location where item is reported
Availability of data, code and other materials	27	Report which of the following are publicly available and where they can be found: template data collection forms; data extracted from included studies; data used for all analyses; analytic code; any other materials used in the review.	Results, Supplementary materials

From: Page MJ, McKenzie JE, Bossuyt PM, Boutron I, Hoffmann TC, Mulrow CD, *et al.*. The PRISMA 2020 statement: an updated guideline for reporting systematic reviews. BMJ 2021;372:n71. doi: 10.1136/bmj.n71

For more information, visit: http://www.prisma-statement.org/

Supplementary Table S3. PRISMA 2020 for Abstracts Checklist

Section and Topic	Item #	Checklist item	Reported (Yes/No)
**TITLE**			
Title	1	Identify the report as a systematic review.	Yes
**BACKGROUND**			
Objectives	2	Provide an explicit statement of the main objective(s) or question(s) the review addresses.	Yes
**METHODS**			
Eligibility criteria	3	Specify the inclusion and exclusion criteria for the review.	Yes
Information sources	4	Specify the information sources (e.g. databases, registers) used to identify studies and the date when each was last searched.	Yes
Risk of bias	5	Specify the methods used to assess risk of bias in the included studies.	Yes
Synthesis of results	6	Specify the methods used to present and synthesise results.	Yes
**RESULTS**			
Included studies	7	Give the total number of included studies and participants and summarise relevant characteristics of studies.	Yes
Synthesis of results	8	Present results for main outcomes, preferably indicating the number of included studies and participants for each. If meta-analysis was done, report the summary estimate and confidence/credible interval. If comparing groups, indicate the direction of the effect (i.e. which group is favoured).	Yes
**DISCUSSION**			
Limitations of evidence	9	Provide a brief summary of the limitations of the evidence included in the review (e.g. study risk of bias, inconsistency and imprecision).	Yes
Interpretation	10	Provide a general interpretation of the results and important implications.	Yes
**OTHER**			
Funding	11	Specify the primary source of funding for the review.	Yes
Registration	12	Provide the register name and registration number.	Yes

From: Page MJ, McKenzie JE, Bossuyt PM, Boutron I, Hoffmann TC, Mulrow CD, *et al.*. The PRISMA 2020 statement: an updated guideline for reporting systematic reviews. BMJ 2021;372:n71. doi: 10.1136/bmj.n71

References

1. Abichabki, N.; Zacharias, L.V.; Moreira, N.C.; Bellissimo-Rodrigues, F.; Moreira, F.L.; Benzi, J.R.L.; Ogasawara, T.M.C.; Ferreira, J.C.; Ribeiro, C.M.; Pavan, F.R.; et al. Potential cannabidiol (CBD) repurposing as antibacterial and promising therapy of CBD plus polymyxin B (PB) against PB-resistant gram-negative bacilli. *Scientific Reports* **2022**, *12*, 6454, doi:10.1038/s41598-022-10393-8.
2. Blaskovich, M.A.T.; Kavanagh, A.M.; Elliott, A.G.; Zhang, B.; Ramu, S.; Amado, M.; Lowe, G.J.; Hinton, A.O.; Pham, D.M.T.; Zuegg, J.; et al. The antimicrobial potential of cannabidiol. *Communications biology* **2021**, *4*, 7, doi:10.1038/s42003-020-01530-y.
3. Farha, M.A.; El-Halfawy, O.M.; Gale, R.T.; MacNair, C.R.; Carfrae, L.A.; Zhang, X.; Jentsch, N.G.; Magolan, J.; Brown, E.D. Uncovering the Hidden Antibiotic Potential of Cannabis. *ACS infectious diseases* **2020**, *6*, 338-346, doi:10.1021/acsinfecdis.9b00419.
4. Kaur, S.; Sharma, C.; Chaudhry, S.; Aman, R. Antimicrobial potential of three common weeds of kurukshetra: An in vitro study. *Research Journal of Microbiology* **2015**, *10*, 280-287, doi:10.3923/jm.2015.280.287.
5. Wassmann, C.S.; Hojrup, P.; Klitgaard, J.K. Cannabidiol is an effective helper compound in combination with bacitracin to kill Gram-positive bacteria. *Scientific Reports* **2020**, *10*, 4112, doi:10.1038/s41598-020-60952-0.
6. Appendino, G.; Gibbons, S.; Giana, A.; Pagani, A.; Grassi, G.; Stavri, M.; Smith, E.; Rahman, M. Antibacterial cannabinoids from *Cannabis sativa*: A structure-activity study. *Journal of Natural Products* **2008**, *71*, 1427-1430, doi:10.1021/np8002673.
7. Chauhan, N.; Farooq, U.; Khan, M.A. Efficacy of medicinal plants against human pathogens isolated from western Himalayas of Himachal Pradesh. *Asian Journal of Pharmaceutical and Clinical Research* **2017**, *10*, 353-357, doi:10.22159/ajpcr.2017.v10i9.19708.
8. Eisohly, H.N.; Turner, C.E.; Clark, A.M.; Eisohly, M.A. Synthesis and antimicrobial activities of certain cannabichromene and cannabigerol related compounds. *Journal of pharmaceutical sciences* **1982**, *71*, 1319-1323, doi:10.1002/jps.2600711204.
9. Muscarà, C.; Smeriglio, A.; Trombetta, D.; Mandalari, G.; La Camera, E.; Grassi, G.; Circosta, C. Phytochemical characterization and biological properties of two standardized extracts from a non-psychotropic *Cannabis sativa* L. cannabidiol (CBD)-chemotype. *Phytotherapy Research* **2021**, *35*, 5269-5281, doi:10.1002/ptr.7201.
10. Muscarà, C.; Smeriglio, A.; Trombetta, D.; Mandalari, G.; La Camera, E.; Occhiuto, C.; Grassi, G.; Circosta, C. Antioxidant and antimicrobial activity of two standardized extracts from a new Chinese accession of non-psychotropic Cannabis sativa L. *Phytotherapy Research* **2021**, *35*, 1099-1112, doi:10.1002/ptr.6891.
11. Turner, C.E.; Elsohly, M.A. Biological activity of cannabichromene, its homologs and isomers. *Journal of Clinical Pharmacology* **1981**, *21*, 283S-291S, doi:10.1002/j.1552-4604.1981.tb02606.x.
12. Van Klingeren, B.; Ten Ham, M. Antibacterial activity of delta9-tetrahydrocannabinol and cannabidiol. *Antonie Van Leeuwenhoek* **1976**, *42*, 9-12, doi:10.1007/BF00399444.

13. Frassinetti, S.; Gabriele, M.; Moccia, E.; Longo, V.; Di Gioia, D. Antimicrobial and antibiofilm activity of *Cannabis sativa* L. seeds extract against *Staphylococcus aureus* and growth effects on probiotic *Lactobacillus* spp. *Lwt-Food Science and Technology* **2020**, *124*, 109149, doi:10.1016/j.lwt.2020.109149.
14. Galletta, M.; Reekie, T.A.; Nagalingam, G.; Bottomley, A.L.; Harry, E.J.; Kassiou, M.; Triccas, J.A. Rapid Antibacterial Activity of Cannabichromenic Acid against Methicillin-Resistant Staphylococcus aureus. *Antibiotics (Basel)* **2020**, *9*, 523, doi:10.3390/antibiotics9080523.
15. Martinenghi, L.D.; Jonsson, R.; Lund, T.; Jenssen, H. Isolation, purification, and antimicrobial characterization of cannabidiolic acid and cannabidiol from *Cannabis sativa* L. *Biomolecules* **2020**, *10*, 900, doi:10.3390/biom10060900.
16. Nafis, A.; Kasrati, A.; Jamali, C.A.; Mezrioui, N.; Setzer, W.; Abbad, A.; Hassani, L. Antioxidant activity and evidence for synergism of *Cannabis sativa* (L.)essential oil with antimicrobial standards. *Industrial Crops and Products* **2019**, *137*, 396-400, doi:10.1016/j.indcrop.2019.05.032.
17. Nigro, E.; Pecoraro, M.T.; Formato, M.; Piccolella, S.; Ragucci, S.; Mallardo, M.; Russo, R.; Di Maro, A.; Daniele, A.; Pacifico, S. Cannabidiolic Acid in Hemp Seed Oil Table Spoon and Beyond. *Molecules* **2022**, *27*, 2566, doi:10.3390/molecules27082566.
18. Gibbons, S. Anti-staphylococcal plant natural products. *Nat Prod Rep* **2004**, *21*, 263-277, doi:10.1039/b212695h.
19. Subramani, R.; Narayanasamy, M.; Feussner, K.D. Plant-derived antimicrobials to fight against multi-drug-resistant human pathogens. *3 Biotech* **2017**, *7*, 172, doi:10.1007/s13205-017-0848-9.
20. Beoletto, V.; De Las Mercedes Oliva, M.; Marioli, J.; Carezzano, M.; Demo, M. Antimicrobial natural products against bacterial biofilms. *Antibiot. Resist* **2016**, *14*, 291-307.
21. Silver, L.; Bostian, K. Screening of natural products for antimicrobial agents. *Eur J Clin Microbiol Infect Dis* **1990**, *9*, 455-461, doi:10.1007/bf01964283.
22. Schofs, L.; Sparo, M.D.; Sánchez Bruni, S.F. The antimicrobial effect behind *Cannabis sativa*. *Pharmacology Research & Perspectives* **2021**, *9*, e00761, doi:10.1002/prp2.761.
23. Klahn, P. Cannabinoids-Promising Antimicrobial Drugs or Intoxicants with Benefits? *Antibiotics-Basel* **2020**, *9*, doi:10.3390/antibiotics9060297.
24. Chouhan, S.; Sharma, K.; Guleria, S. Antimicrobial Activity of Some Essential Oils-Present Status and Future Perspectives. *Medicines (Basel)* **2017**, *4*, 58, doi:10.3390/medicines4030058.
25. Fathordoobady, F.; Singh, A.; Kitts, D.D.; Pratap Singh, A. Hemp (*Cannabis Sativa* L.) Extract: Anti-Microbial Properties, Methods of Extraction, and Potential Oral Delivery. *Food Reviews International* **2019**, *35*, 664-684, doi:10.1080/87559129.2019.1600539.
26. Glodowska, M.; Łyszcz, M. *Cannabis sativa* L. and its antimicrobial properties – A review. In Proceedings of the Badania i Rozwój Młodych Naukowców w Polsce – Agronomia i ochrona roślin, 2016; pp. 77-82.

27. Tandon, C.; Mathur, P. Antimicrobial Efficacy of Cannabis sativa L. (Bhang): A review. *Int. J. Pharm. Sci. Rev. Res.* **2017**, *44*, 94-100.
28. Iseppi, R.; Brighenti, V.; Licata, M.; Lambertini, A.; Sabia, C.; Messi, P.; Pellati, F.; Benvenuti, S. Chemical Characterization and Evaluation of the Antibacterial Activity of Essential Oils from Fibre-Type *Cannabis sativa* L. (Hemp). *Molecules* **2019**, *24*, 2302, doi:http://dx.doi.org/10.3390/molecules24122302.
29. Ali, E.M.M.; Almagboul, A.Z.I.; Khogali, S.M.E.; Gergeir, U.M.A. Antimicrobial activity of *Cannabis sativa* L. *Chinese Medicine* **2012**, *3*, 61-64, doi:10.4236/cm.2012.31010.
30. Sarmadyan, H.; Solhi, H.; Hajimir, T.; Najarian-Araghi, N.; Ghaznavi-Rad, E. Determination of the antimicrobial effects of hydro-alcoholic extract of *Cannabis sativa* on multiple drug resistant bacteria isolated from nosocomial infections. *Iranian Journal of Toxicology* **2014**, *7*, 967-972.
31. Vu, T.T.; Kim, H.; Tran, V.K.; Le Dang, Q.; Nguyen, H.T.; Kim, H.; Kim, I.S.; Choi, G.J.; Kim, J.C. In vitro antibacterial activity of selected medicinal plants traditionally used in Vietnam against human pathogenic bacteria. *BMC Complement Altern Med* **2016**, *16*, 32, doi:10.1186/s12906-016-1007-2.
32. Zengin, G.; Menghini, L.; Sotto, A.D.; Mancinelli, R.; Sisto, F.; Carradori, S.; Cesa, S.; Fraschetti, C.; Filippi, A.; Angiolella, L.; et al. Chromatographic analyses, in vitro biological activities, and cytotoxicity of *Cannabis sativa* L. Essential oil: A multidisciplinary study. *Molecules* **2018**, *23*, 3266, doi:10.3390/molecules23123266.
33. ACTRN12620000456954. A Randomised, Double-Blind, Vehicle-Controlled Study to Evaluate Safety, Tolerability, and Efficacy of Two Dosage Forms of BTX 1801 Applied Twice Daily for Five Days to the Anterior Nares of Healthy Adults Nasally Colonised with Staphylococcus aureus. *https://trialsearch.who.int/Trial2.aspx?TrialID=ACTRN12620000456954* **2020** (accessed on 28 May 2024).
34. Feldman, M.; Smoum, R.; Mechoulam, R.; Steinberg, D. Antimicrobial potential of endocannabinoid and endocannabinoid-like compounds against methicillin-resistant Staphylococcus aureus. *Scientific Reports* **2018**, *8*, 17696, doi:10.1038/s41598-018-35793-7.
35. Pochay, V.; McCarthy, C.; Pereira, W. Impairment of antibacterial defenses of the lung by marijuana smoke. *Clinical Research* **1975**, *23*, 599A.
36. Napiroon, T.; Tanruean, K.; Poolprasert, P.; Bacher, M.; Balslev, H.; Poopath, M.; Santimaleeworagun, W. Cannabinoids from inflorescences fractions of *Trema orientalis* (L.) Blume (Cannabaceae) against human pathogenic bacteria. *PeerJ* **2021**, *9*, e11446, doi:10.7717/peerj.11446.
37. Feldman, M.; Smoum, R.; Mechoulam, R.; Steinberg, D. Potential combinations of endocannabinoid/endocannabinoid-like compounds and antibiotics against methicillin-resistant *Staphylococcus aureus*. *PLoS One* **2020**, *15*, e0231583, doi:10.1371/journal.pone.0231583.
38. Nasrullah; Suliman; Rahman, K.; Ikram, M.; Nisar, M.; Khan, I. Screening of antibacterial activity of medicinal plants. *International Journal of Pharmaceutical Sciences Review and Research* **2012**, *14*, 25-29.

39. Radwan, M.M.; Ross, S.A.; Slade, D.; Ahmed, S.A.; Zulfiqar, F.; ElSohly, M.A. Isolation and characterization of new cannabis constituents form high potency variety. *Planta Medica* **2008**, *74*, 267-272, doi:10.1055/s-2008-1034311.
40. Nissen, L.; Zatta, A.; Stefanini, I.; Grandi, S.; Sgorbati, B.; Biavati, B.; Monti, A. Characterization and antimicrobial activity of essential oils of industrial hemp varieties (*Cannabis sativ*a L.). *Fitoterapia* **2010**, *81*, 413-419, doi:10.1016/j.fitote.2009.11.010.
41. Das, B.; Mishra, P.C. Antibacterial analysis of crude extracts from the leaves of Tagetes erecta and Cannabis sativa. *Agris On-line Papers in Economics and Informatics* **2012**, *2*, 1605-1609, doi:10.6088/ijes.002020300045.
42. Elhendawy, M.A.; Wanas, A.S.; Radwan, M.M.; Azzaz, N.A.; Toson, E.S.; Elsohly, M.A. Chemical and Biological Studies of Cannabis sativa Roots. *Med. Cannabis Cannabinoids* **2019**, *1*, 104-111, doi:10.1159/000495582.
43. Pereira, W.; McLaughlin, T.; Baranano, M.T. The acute effect of marijuana smoke on antibacterial defense mechanisms of the lung. *Clinical Research* **1975**, *23*, 351.
44. Ahmed, S.A.; Ibrahim, A.K.; Radwan, M.M.; Slade, D.; Chandra, S.; Khan, I.A.; Elsohly, M.A. Microbial Biotransformation of Cannabidiol (CBD) from Cannabis sativa. *Planta Medica* **2022**, *88*, 389-397, doi:10.1055/a-1468-3781.
45. Appendino, G.; Giana, A.; Gibbons, S.; Maffei, M.; Gnavi, G.; Grassi, G.; Sterner, O. A Polar Cannabinoid from Cannabis sativa var. Carma. *Natural Product Communications* **2008**, *3*, 1977-1980.
46. Kumar, V.; Tripathi, M.K.; Kohli, S. Antibacterial activity of Cannabis sativa against some pathogens isolated from burns of patient. *Medicinal Plants* **2011**, *3*, 243-247, doi:10.5958/j.0975-4261.3.3.039.
47. Cortes, E.; Mora, J.; Márquez, E. Modelling the anti-methicillin-resistant staphylococcus aureus (MRSA) activity of cannabinoids: A QSAR and docking study. *Crystals* **2020**, *10*, 1-20, doi:10.3390/cryst10080692.
48. Gür, M.; Verep, D.; Güney, K.; Güder, A.; Altuner, E.M. Determination of some flavonoids and antimicrobial behaviour of some plants' extracts. *Indian Journal of Pharmaceutical Education and Research* **2017**, *51*, S225-S229, doi:10.5530/ijper.51.3s.18.
49. Radwan, M.M.; Elsohly, M.A.; Slade, D.; Ahmed, S.A.; Wilson, L.; El-Alfy, A.T.; Khan, I.A.; Ross, S.A. Non-cannabinoid constituents from a high potency Cannabis sativa variety. *Phytochemistry* **2008**, *69*, 2627-2633, doi:10.1016/j.phytochem.2008.07.010.
50. Wassmann, C.S.; Rolsted, A.P.; Lyngsie, M.C.; Torres-Puig, S.; Kronborg, T.; Vestergaard, M.; Ingmer, H.; Pontoppidan, S.P.; Klitgaard, J.K. The menaquinone pathway is important for susceptibility of Staphylococcus aureus to the antibiotic adjuvant, cannabidiol. *Microbiological research* **2022**, *257*, 126974, doi:10.1016/j.micres.2022.126974.
51. Menjivar, J.; Bendaoud, M. Anti-Biofilm Properties of Flax, Chia, and Hemp Seed Oil Extracts. *Faseb Journal* **2020**, *34*, 1, doi:10.1096/fasebj.2020.34.s1.05146.
52. Shah, S.B.; Sartaj, L.; Hussain, S.; Ullah, N.; Idrees, M.; Shaheen, A.; Javed, M.S.; Aslam, M.K. In-vitro evaluation of antimicrobial, antioxidant, alpha-amylase inhibition and cytotoxicity properties of Cannabis sativa. *Advances in Traditional Medicine* **2020**, *20*, 181-187, doi:10.1007/s13596-019-00414-9.

53. Mkpenie, V.N.; Essien, E.E.; Udoh, I.I. Effect of extraction conditions on total polyphenol contents, antioxidant and antimicrobial activities of Cannabis sativa L. *Electronic Journal of Environmental, Agricultural and Food Chemistry* **2012**, *11*, 300-307.
54. Kostic, M.M.; Milanovic, J.Z.; Baljak, M.V.; Mihajlovski, K.; Kramar, A.D. Preparation and characterization of silver-loaded hemp fibers with antimicrobial activity. *Fibers and Polymers* **2014**, *15*, 57-64, doi:10.1007/s12221-014-0057-7.
55. Ahmed, S.A.; Ross, S.A.; Slade, D.; Radwan, M.M.; Khan, I.A.; Elsohly, M.A. Structure determination and absolute configuration of cannabichromanone derivatives from high potency Cannabis sativa. *Tetrahedron letters* **2008**, *49*, 6050-6053, doi:10.1016/j.tetlet.2008.07.178.
56. Ali, M.; Romman, M.; Parvez, R.; Shuaib, M.; Bahadur, S.; Khalil, A.A.K.; Khan, M.; ul Haq, F.; Jan, S.; Hayat, S.S.S.; et al. Anti-bacterial activity of Cannabis sativa Linn. leaf extracts against different pathogenic bacterial strains. *Bioscience Research* **2020**, *17*, 2730-2735.
57. Chakraborty, S.; Afaq, N.; Singh, N.; Majumdar, S. Antimicrobial activity of Cannabis sativa, Thuja orientalis and Psidium guajava leaf extracts against methicillin-resistant Staphylococcus aureus. *Journal of integrative medicine* **2018**, *16*, 350-357, doi:10.1016/j.joim.2018.07.005.
58. Ferenczy, L.; Gracza, L.; Jakobey, I. An antibacterial preparatum from hemp (Cannabis sativa L.). *Die Naturwissenschaften* **1958**, *45*, 188, doi:10.1007/BF00621336.
59. Verma, R.S.; Padalia, R.C.; Verma, S.K.; Chauhan, A.; Darokar, M.P. The essential oil of 'bhang' (Cannabis sativa L.) for non-narcotic applications. *Current Science* **2014**, *107*, 645-650.
60. Novak, J.; Zitterl-Eglseer, K.; Deans, S.G.; Franz, C.M. Essential oils of different cultivars of Cannabis sativa L. and their antimicrobial activity. *Flavour and Fragrance Journal* **2001**, *16*, 259-262, doi:10.1002/ffj.993.
61. Wasim, K.; Haq, I.; Ashraf, M. Antimicrobial studies of the leaf of cannabis sativa L. *Pakistan journal of pharmaceutical sciences* **1995**, *8*, 29-38.
62. Chauhan, A.; Verma, R.; Kumari, S.; Sharma, A.; Shandilya, P.; Li, X.; Batoo, K.M.; Imran, A.; Kulshrestha, S.; Kumar, R. Photocatalytic dye degradation and antimicrobial activities of Pure and Ag-doped ZnO using Cannabis sativa leaf extract. *Sci Rep* **2020**, *10*, 7881, doi:10.1038/s41598-020-64419-0.
63. Radwan, M.M.; Elsohly, M.A.; Slade, D.; Ahmed, S.A.; Khan, I.A.; Ross, S.A. Biologically active cannabinoids from high-potency Cannabis sativa. *Journal of Natural Products* **2009**, *72*, 906-911, doi:10.1021/np900067k.
64. Hazrat, A.; Nisar, M.; Zaman, S. ntibacterial activities of sixteen species of medicinal plants reported from Dir Kohistan valley kpk, Pakistan. *Pakistan Journal of Botany* **2013**, *45*, 1369-1374.
65. Manosroi, A.; Chankhampan, C.; Kietthanakorn, B.-o.; Ruksiriwanich, W.; Chaikul, P.; Boonpisuttinant, K.; Sainakham, M.; Manosroi, W.; Tangjai, T.; Manosroi, J. Pharmaceutical and Cosmeceutical Biological Activities of Hemp (Cannabis sativa L var. sativa) Leaf and Seed Extracts. *Chiang Mai Journal of Science* **2019**, *46*, 180-195.

66. Pasquali, F.; Schinzari, M.; Lucchi, A.; Mandrioli, M.; Toschi, T.G.; De Cesare, A.; Manfreda, G. Preliminary data on the antimicrobial effect of Cannabis sativa L. variety Futura 75 against food-borne pathogens in vitro as well as against naturally occurring microbial populations on minced meat during storage. *Italian Journal of Food Safety* **2020**, *9*, 80-87, doi:10.4081/ijfs.2020.8581.
67. Zheljazkov, V.D.; Sikora, V.; Dincheva, I.; Kacaniova, M.; Astatkie, T.; Semerdjieva, I.B.; Latkovic, D. Industrial, CBD, and Wild Hemp: How Different Are Their Essential Oil Profile and Antimicrobial Activity? *Molecules* **2020**, *25*, 4631, doi:10.3390/molecules25204631.
68. Barbălată-Mândru, M.; Serbezeanu, D.; Butnaru, M.; Rîmbu, C.M.; Enache, A.A.; Aflori, M. Poly(vinyl alcohol)/Plant Extracts Films: Preparation, Surface Characterization and Antibacterial Studies against Gram Positive and Gram Negative Bacteria. *Materials* **2022**, *15*, 2493, doi:10.3390/ma15072493.
69. Mikulcová, V.; Kašpárková, V.; Humpolíček, P.; Buňková, L. Formulation, Characterization and Properties of Hemp Seed Oil and Its Emulsions. *Molecules (Basel, Switzerland)* **2017**, *22*, 700, doi:10.3390/molecules22050700.
70. Rao, R.; Nagarkatti, P.S.; Nagarkatti, M. Delta(9)Tetrahydrocannabinol attenuates Staphylococcal enterotoxin B-induced inflammatory lung injury and prevents mortality in mice by modulation of miR-17-92 cluster and induction of T-regulatory cells. *British Journal of Pharmacology* **2015**, *172*, 1792-1806, doi:10.1111/bph.13026.
71. Ashfaq, M.K.; Watson, E.S.; elSohly, H.N. The effect of subacute marijuana smoke inhalation on experimentally induced dermonecrosis by S. aureus infection. *Immunopharmacology and immunotoxicology* **1987**, *9*, 319-331, doi:10.3109/08923978709035217.
72. Huber, G.L.; Pochay, V.E.; Pereira, W.; Huber, G.L.; Pochay, V.E.; Pereira, W.; Shea, J.W.; Hinds, W.C.; First, M.W.; Sornberger, G.C. Marijuana, tetrahydrocannabinol, and pulmonary antibacterial defenses. *Chest* **1980**, *77*, 403-410.
73. Zhong, G.; Tong, C.; Liu, X.; Fan, J.; Xiong, X.; Chen, P.; Zhu, A.; Yu, R.; Liu, B. Chi@HMPB@CBD nanocomplexes for laser-assisted therapy of MRSA-infected cutaneous wounds in normal and MKR diabetic mice. *Mater. Today Chem.* **2022**, *24*, 100888, doi:10.1016/j.mtchem.2022.100888.
74. Russo, C.; Lavorgna, M.; Nugnes, R.; Orlo, E.; Isidori, M. Comparative assessment of antimicrobial, antiradical and cytotoxic activities of cannabidiol and its propyl analogue cannabidivarin. *Scientific Reports* **2021**, *11*, 22494, doi:10.1038/s41598-021-01975-z.
75. Dudley, A.; Kassama, L.; Jackson-Davis, A.; Cebert, E.; Mohammaed, A. In vitro evaluation of antibacterial and antioxidative activity of two northern Alabama hemp (Cannabis sativa L) varieties. In Proceedings of the American Society of Agricultural and Biological Engineers Annual International Meeting, ASABE 2021, 2021; pp. 2343-2354.
76. Fournier, G.; Paris, M.R.; Fourniat, M.C.; Quero, A.M. [Bacteriostatic activity of *Cannabis sativa* L. essential oil (author's transl)]. *Annales Pharmaceutiques Francaises* **1978**, *36*, 603-606.
77. Frankova, A.; Janatova, A.; Tauchen, J.; Kokoska, L. In vitro Antimicrobial and Antioxidant Activity of Extracts from Six Chemotypes of Medicinal Cannabis. *Planta Medica* **2016**, *82*, S1-S381, doi:10.1055/s-0036-1596302.

78. Ujváry, I.; Hanuš, L. Human Metabolites of Cannabidiol: A Review on Their Formation, Biological Activity, and Relevance in Therapy. *Cannabis Cannabinoid Res* **2016**, *1*, 90-101, doi:10.1089/can.2015.0012.
79. Tahir, M.N.; Shahbazi, F.; Rondeau-Gagné, S.; Trant, J.F. The biosynthesis of the cannabinoids. *Journal of Cannabis Research* **2021**, *3*, 7, doi:10.1186/s42238-021-00062-4.
80. Typek, R.; Holowinski, P.; Dawidowicz, A.L.; Dybowski, M.P.; Rombel, M. Chromatographic analysis of CBD and THC after their acylation with blockade of compound transformation. *Talanta* **2023**, *251*, 123777, doi:https://doi.org/10.1016/j.talanta.2022.123777.
81. Consroe, P.; Martin, A.; Singh, V. Antiepileptic Potential of Cannabidiol Analogs. *The Journal of Clinical Pharmacology* **1981**, *21*, 428S-436S, doi:https://doi.org/10.1002/j.1552-4604.1981.tb02623.x.
82. Pertwee, R.G.; Rock, E.M.; Guenther, K.; Limebeer, C.L.; Stevenson, L.A.; Haj, C.; Smoum, R.; Parker, L.A.; Mechoulam, R. Cannabidiolic acid methyl ester, a stable synthetic analogue of cannabidiolic acid, can produce 5-HT(1A) receptor-mediated suppression of nausea and anxiety in rats. *Br J Pharmacol* **2018**, *175*, 100-112, doi:10.1111/bph.14073.
83. Anderson, L.L.; Ametovski, A.; Lin Luo, J.; Everett-Morgan, D.; McGregor, I.S.; Banister, S.D.; Arnold, J.C. Cannabichromene, Related Phytocannabinoids, and 5-Fluoro-cannabichromene Have Anticonvulsant Properties in a Mouse Model of Dravet Syndrome. *ACS Chemical Neuroscience* **2021**, *12*, 330-339, doi:10.1021/acschemneuro.0c00677.
84. Nguyen, G.-N.; Jordan, E.N.; Kayser, O. Synthetic Strategies for Rare Cannabinoids Derived from *Cannabis sativa*. *Journal of Natural Products* **2022**, *85*, 1555-1568, doi:10.1021/acs.jnatprod.2c00155.
85. Pellegrini, M.; Palmieri, S.; Ricci, A.; Serio, A.; Paparella, A.; Lo Sterzo, C. In vitro antioxidant and antimicrobial activity of *Cannabis sativa* L. cv 'Futura 75' essential oil. *Natural Product Research* **2020**, *35*, 6020-6024, doi:10.1080/14786419.2020.1813139.
86. Pellegrini, M.; Ricci, A.; Serio, A.; Chaves-López, C.; Mazzarrino, G.; D'Amato, S.; Lo Sterzo, C.; Paparella, A. Characterization of essential oils obtained from Abruzzo autochthonous plants: Antioxidant and antimicrobial activities assessment for food application. *Foods* **2018**, *7*, 19, doi:10.3390/foods7020019.
87. Schuetz, M.; Savile, C.; Webb, C.; Rouzard, K.; Fernandez, J.R.; Perez, E. Cannabigerol: The mother of cannabinoids demonstrates a broad spectrum of anti-inflammatory and anti-microbial properties important for skin. *Journal of Investigative Dermatology* **2021**, *141*, S83-S83.
